# Nanoscale Mapping Reveals Periodic Organization of Neutrophil Extracellular Trap Proteins

**DOI:** 10.1021/acs.nanolett.5c05175

**Published:** 2026-04-01

**Authors:** Moritz Winkler, Till G. A. Mack, Britta J. Eickholt, Jan Schmoranzer, Garth Lawrence Burn, Niclas Gimber

**Affiliations:** † Department of Cellular Microbiology, 28260 Max Planck Institute for Infection Biology, 10117 Berlin, Germany; ‡ Advanced Medical Bioimaging Core Facility (AMBIO), 14903 Charité-Universitätsmedizin Berlin, 10117 Berlin, Germany; § Institute of Biochemistry and Molecular Biology, Charité-Universitätsmedizin Berlin, 10117 Berlin, Germany

**Keywords:** Neutrophil extracellular traps, innate immunity, super-resolution microscopy, single-molecule localization microscopy, structured illumination microscopy, stimulated emission depletion microscopy

## Abstract

Neutrophils are essential cells of the innate immune system that release neutrophil extracellular traps (NETs) via NETosis. This specialized cell death pathway extrudes decondensed chromatin into the extracellular space. NET formation contributes to antimicrobial defense and coagulation, whereas dysregulated NETosis drives cancer, coagulopathy, and autoimmune diseases. Here we present a workflow combining optimized sample preparation, super-resolution imaging, and quantitative bioimage analysis to investigate the nanoscale organization of NETs. Our newly developed analysis tool, NanoNET, facilitates the NET protein binding pattern analysis. We identified specific NET proteins, including neutrophil elastase (NE) and proteinase 3 (PR3), that bind along DNA strands in a periodic fashion and are highly colocalized with nucleosomes, suggesting docking onto or around these structures. The workflow and analysis tools represent a significant methodological advance for studying protein distributions along NETs and filamentous structures in general. Understanding the NET filament organization is a critical step toward elucidating their formation and function.

## Graphical abstract

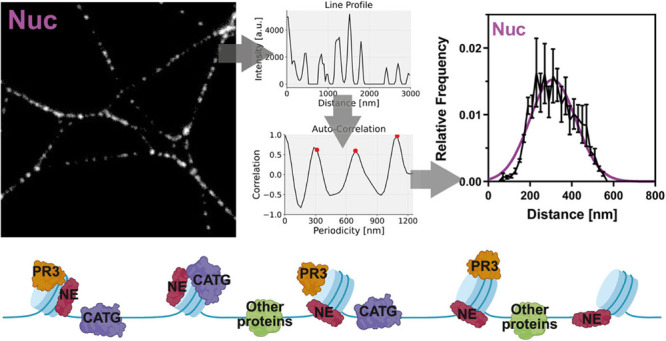

Neutrophils play a pivotal role in innate immunity during septic and aseptic injury.^1,2^ One key effector mechanism of neutrophils is the formation of neutrophil extracellular traps (NETs) via a cell death program called NETosis.^3^ During NETosis, nuclear chromatin is decondensed and transformed into cell-free scaffolds by the cytoplasmic effector proteins. These atypical chromatin structures contain modified nucleosomes and are decorated with a distinct set of cytoplasmic proteins, most of which are immune-related.^4,5^ Both exogenous and endogenous stimuli can lead to NETosis.^6,7^ Once initiated, NET formation proceeds via several discrete yet overlapping processes which include the translocation of cytoplasmic proteins into the nucleus, nuclear envelope breakdown, the intermixing of decondensed chromatin with the cytoplasm, and finally, the rupturing of the plasma membrane which releases NETs into the extracellular space.^8^ When NETs are released appropriately, they play an important role in maintaining homeostasis by trapping and killing microbes or initiating coagulation, however, the unbridled release of NETs is associated with cancer, coagulopathy, and autoimmune disease.^9^ Despite the growing clinical relevance of NETs, the underlying nanoscale organization of NET proteins along extruded DNA filaments remains largely unknown.

To investigate whether NET proteins bind in distinct nanoscale patterns that cannot be resolved using conventional light microscopy, we used three different complementary super-resolution techniques.^10^ A key challenge was to avoid DNA filament aggregation and to identify individual filaments. To efficiently screen large numbers of filaments for patterned NET protein distributions, we developed NanoNET, an open-source, automated toolbox for analyzing nanoscale patterns along NET filaments. Using NanoNET, we were able to identify individual DNA strands and subsequently investigate periodic binding patterns of different NET-associated proteins.

Understanding the nanoscale organization of proteins on NETs is a key step toward unraveling their nanoscale architecture and potential structure–function relationships that underpin NET biology.

## Imaging and Analysis Workflow for Neutrophil Extracellular Traps

To enable reliable imaging of individual NET filaments without excessive DNA aggregation or disruption of the nanostructure, we have developed a protocol that safeguards DNA filaments from extensive aggregation and makes them compatible with super-resolution imaging (Figure 1A). After freshly isolated neutrophils were seeded onto coverslips, NET formation was induced using the mitogen phorbol 12-myristate 13-acetate (PMA), followed by a specific fixation protocol and mounting procedure. The resulting NET structures were highly planar, making them ideal for super-resolution imaging (Figure S1). A detailed description of the protocol is provided in Methods. We used the DNA intercalating dye YOYO-1 to stain the NET backbone due to its high quantum yield and excellent signal-to-noise ratio, making this dye ideal for NET detection (Figures 1B and S1). To automate the analysis of thousands of uniformly sized NET fragments, we developed the NanoNET Toolbox (Figure 1C; also see https://github.com/ngimber/NanoNET), which efficiently segments DNA strands, generates line profiles, and produces auto- and cross-correlation plots for NET-associated proteins (Figure 1B–D). Using antibodies directed against NET-specific proteins, we were able to analyze the periodicity and colocalization of those proteins along the DNA-labeled NET filaments. Utilizing three different super-resolution microscopy techniques, and by generating auto- and cross-correlation histograms, we demonstrate here that a subset of highly abundant^4^ NET binding proteins are periodically distributed and colocalize with nucleosomes.

**Figure 1:**
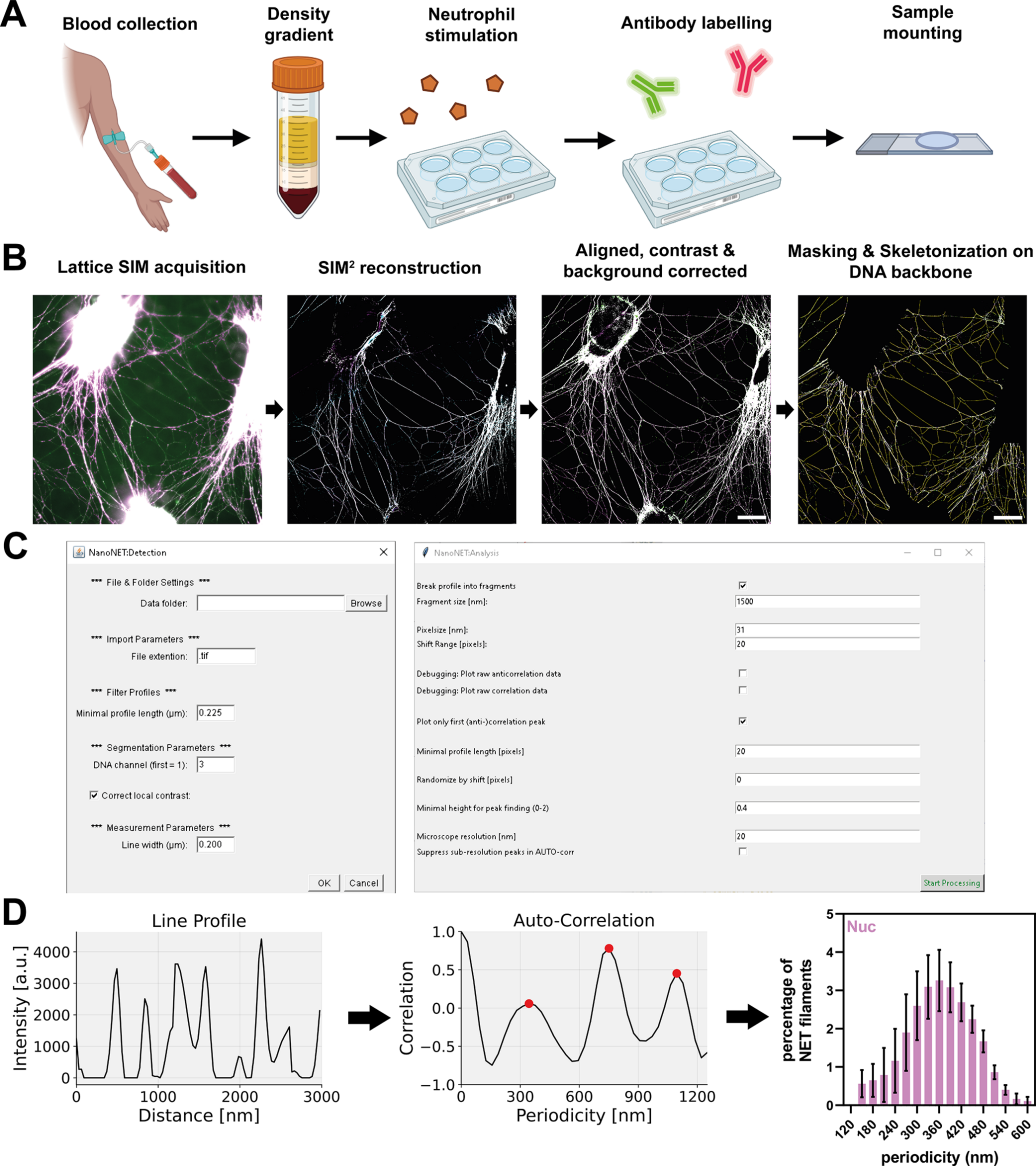
**Nanoscale protein mapping of NET samples.** (A) Schematic of NET preparation for super-resolution microscopy. Neutrophils were purified by a two-step density gradient from fresh blood samples collected from healthy donors. NET formation was stimulated with PMA on glass coverslips followed by fixation, antibody labeling and sample mounting. (Details are provided in Methods). Illustrations were created with BioRender. (B) Imaging and processing pipeline. SIM images were acquired and reconstructed using the Zeiss SIM^2^ module. Multicolor channels were aligned and images were background-corrected before generating maximum intensity projections. Regions with single thin filaments were automatically processed using “NanoNET-Detection” for the detection of single NET filaments based on DNA signal. Scale bar = 10 μm. (C) NanoNET toolbox for automated NET-Detection (left) and NET-Analysis (right). (D) “NanoNET-Analysis” was used to extract intensity line profiles along skeletonized NET filaments as equally sized fragments (left), to perform the autocorrelation analysis of NET protein signals (middle), and to generate periodicity histograms (right).

## SIM Reveals Periodic Distribution of Nucleosomes on NETs and Distinct Binding Modes of Neutrophil Serine Proteases

We used multichannel structured illumination microscopy (SIM) to investigate the nanoscale organization of NET proteins and nucleosomes (as potential interaction hubs) along NET filaments. SIM provides a 2-fold gain of resolution over conventional (confocal) imaging in both the lateral (*x*, *y*) and axial (*z*) dimensions and allows the use of conventional fluorophores.^11^ Additionally, SIM does not require very high laser powers and offers shorter acquisition times compared to other super-resolution techniques (e.g., SMLM and STED), making this technique perfectly suited to initially screen several NET-associated proteins on the nanoscale while maintaining a medium-to-high throughput.^10^ We first focused on the neutrophil serine proteases (NSP) NE and CATG. NSPs are a set of highly conserved immune cell restricted proteases that are expressed mainly in neutrophils and have been shown to be important immune effectors by cleaving bacterial virulence factors, remodeling extracellular matrix, regulating pro-inflammatory mediators, and, interestingly, are also implicated in NET formation.^12,13^ Both NE and CATG have previously been reported to bind to NETs.^4^ NETs were colabeled with YOYO-1 (DNA backbone), the nucleosome antibody PL2.3 (recognizes a conformational H2A-H2B-DNA epitope), and antibodies against NE and CATG. By applying SIM we found that nucleosomes, NE, and CATG localize as globular puncta along individual NET filaments (Figures 2A and S1), reminiscent of the globular structures seen on NETs by scanning electron microscopy (SEM).^14^ To comprehensively analyze the spatial distribution of NET proteins along the DNA backbone, including tests for potential periodic distributions, we designed the toolbox NanoNET. Using NanoNET allowed us to generate spatial autocorrelograms from a high number of NET protein signals (e.g., nucleosomes, NE, and CATG) along the automatically detected DNA backbone. For each protein, we determined the lag distance of the first peak within the autocorrelogram to generate a periodicity histogram of a large number of individual NET protein profiles (see Methods and Figures 2B,C and S2). Using Gaussian fitting, we extracted the predominant periodicity from the histograms. We found that NE and nucleosomes exhibited similar periodicities of 370 and 383 nm, respectively (Figure 2D), reflecting comparable spacing between individual nanodomains of both proteins along the filamentous meshwork of NETs. Similarly, proteinase 3 (PR3), another NET-binding^4^ NSP family member, displayed a periodicity of 375 nm (Figure S3). In contrast, CATG displayed a weak periodicity (low peak), albeit with a similar spacing as nucleosomes, NE, and PR3. Altogether, this might indicate that NE and PR3 depend on a nearby nucleosome to bind to NETs, whereas CATG follows a different binding scheme.

**Figure 2:**
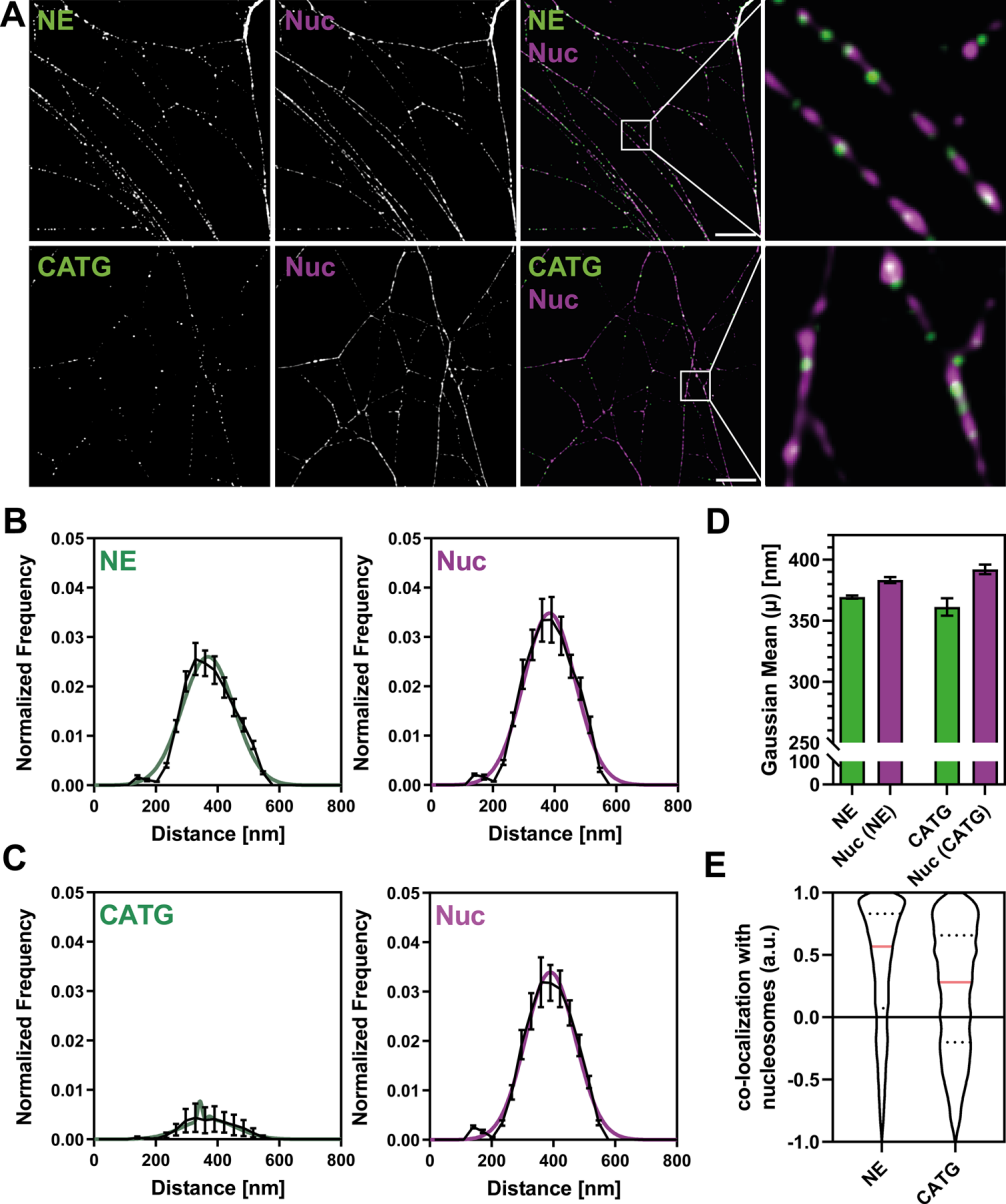
**SIM reveals periodic distribution of nucleosomes on NETs and distinct binding modes of different NSPs.** Colabeling of NSPs with nucleosomes (Nuc, mAB PL2.3) on NETs. Data from five independent donors are shown. Analyzed line profiles (1.5 μm each): NE = 60,420; CATG = 66,205. (A) Representative SIM images of NE (top) and CATG (bottom) colabeled with nucleosomes. Scale bar: 3 μm; inset box: 2 μm × 2 μm. (B, C) Periodicity histograms for NE, CATG, and Nuc along NETs. Averages of Gaussian fits are shown as colored lines. Note the pronounced periodicity of NE and Nuc (prominent peaks), in contrast to CATG, which lacks prominent periodicity. Mean ± SEM shown as black lines. (D) Centers of the Gaussian fits in (b) and (c), reflecting the predominant periodicity of each protein. Mean ± SEM. (E) Cross-correlation analysis of NE or CATG with nucleosomes, replotted from Figure S4. NE shows strong colocalization with nucleosomes, whereas CATG displays a multimodal distribution. Analysis only includes double-labeled NET fragments (NE: 32.6%; CATG: 11.2%). Median ± quartiles.

To test whether all three NSPs colocalize with nucleosomes, we analyzed their cross-correlation with nucleosomes using NanoNET. Our analysis revealed that NE and PR3 were highly cross-correlated/colocalized with nucleosomes, while CATG, in comparison, exhibited a lower cross-correlation with nucleosomes (Figures 2E and S4). These data corroborate our previous findings when comparing the periodicity of NE and PR3 with nucleosomes suggesting that these two proteins directly or indirectly associate with or around nucleosomes, while CATG follows a different binding mode. We validated the periodicity analysis by comparing either two NE antibodies (monoclonal vs polyclonal) or two nucleosome markers (PL2.3 vs 3D9, an antibody against the cleavage site of NET-associated nucleosomes on the H3 tail;^15^ Figure S5). In both conditions, two markers against the same target revealed very similar periodicity in terms of prominence and spacing, demonstrating the robustness of our assay and analysis pipeline. To test whether NET protein spacing depends on the inducing stimulus, we stimulated neutrophils with the bacterial pore-forming toxin Panton–Valentine leukocidin (PVL),^16^ which triggers NET formation via a NADPH oxidase independent pathway, distinct from PMA-induced NETosis.^17,18^ Staining PVL-induced NETs with the antihistone antibody PL2.3 revealed an almost identical histone periodicity to PMA-induced NETs (Figure S6), indicating that this nanoscale organization is preserved despite different upstream mechanisms. In parallel to this project, we studied the distribution of myeloperoxidase (MPO) on NETs biochemically. Using the cross-correlation analysis of NanoNET on MPO and nucleosomes, we confirmed the biochemical finding that myeloperoxidase associates with the nucleosome core complex on NETs,^19^ further validating the method.

Additionally, we applied our spatial analysis to other NET proteins that were recently identified by mass spectrometry. Human neutrophil peptide 1 (HNP-1), catalase (CAT), calgranulin B (S100A9), and transaldolase 1 (TALDO1) displayed no clear periodicity (Figures S7 and S8) and, except for HNP-1, exhibited no significant colocalization with nucleosomes (Figure S4).

Altogether, our results suggest that periodicity is a distinct feature of specific NSPs, particularly NE and PR3, that mirrors the periodicity of nucleosomes in addition to being highly cross-correlated with nucleosomes. Other NET-associated proteins display less periodic signals or do not colocalize readily with nucleosomes. This differential organization may reflect distinct ways in which different proteins interact with the NETs.

## STED Microscopy Confirms Periodic Binding Patterns of NET Proteins

The SIM system we used provides a lateral resolution of ∼100 nm, whereas the available STED setup can resolve structures as small as 50–75 nm. Therefore, we used STED (Figure S9) to validate the periodicity observed with SIM and to search for potentially smaller underlying structures without the need for image reconstruction.^20^ STED images of nucleosomes colabeled with NE or PR3 revealed a highly periodic distribution of both proteins along NET filaments, consistent with the patterns observed using SIM, while CATG showed no obvious periodicity (Figures 3A and S10a). We analyzed the images using NanoNET and obtained periodicity histograms that revealed pronounced peaks for NE, PR3 and nucleosomes, confirming their periodic organization (Figures 3B–D and S10b,c). CATG remained largely aperiodic, in line with the weak periodicity peak in SIM and with STED’s lower sensitivity due to photobleaching (Figure 3C). Both super-resolution methods revealed highly periodic distributions for NE, PR3, and nucleosomes, but a predominantly aperiodic distribution of CATG.

**Figure 3:**
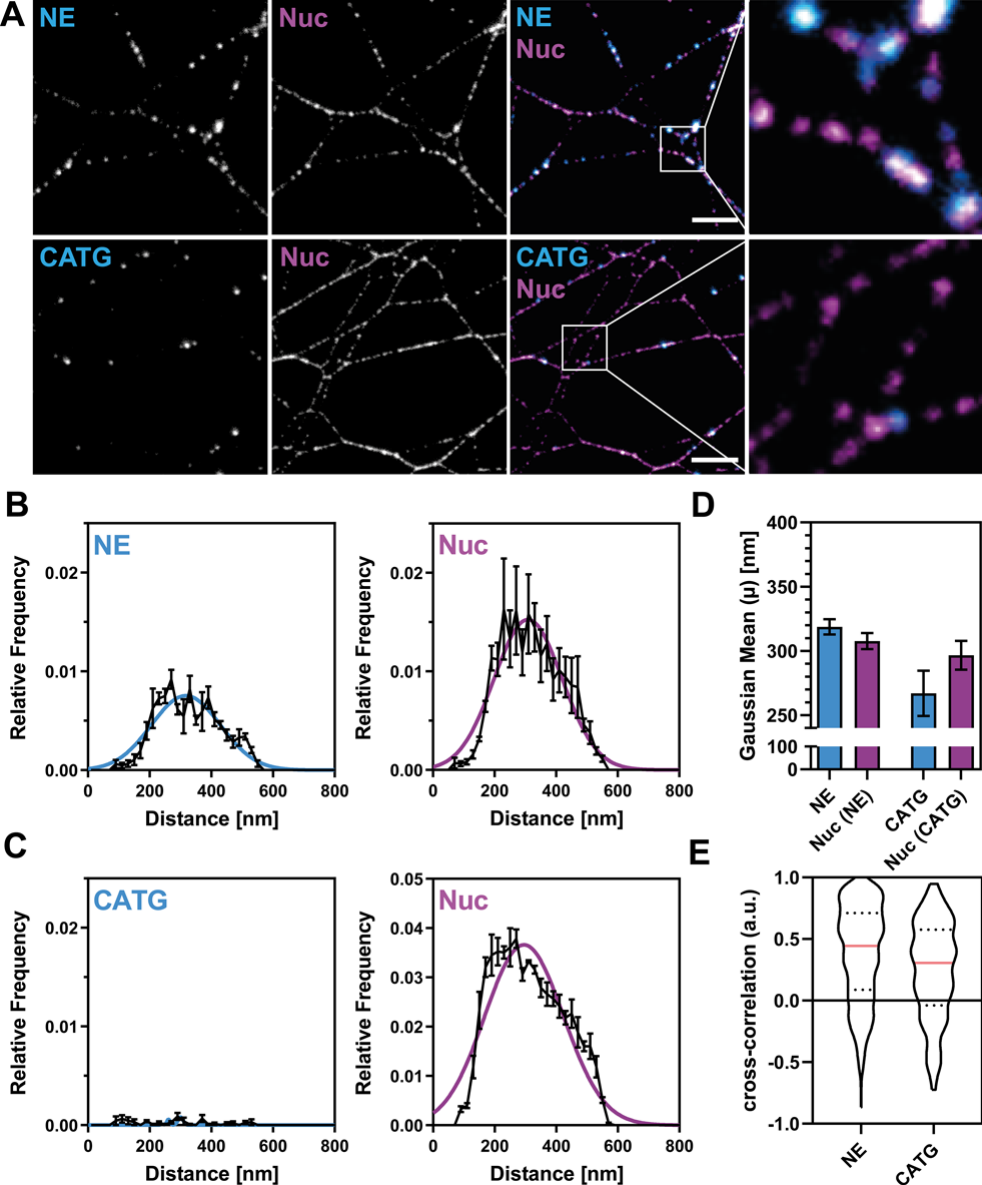
**STED microscopy validates the periodic binding patterns of proteins on NETs and the colocalization of NE and Nuc.** Colabeling of neutrophil serine proteases with nucleosomes (Nuc mAB PL2.3) on NETs. Data from three independent donors are shown. Analyzed line profiles (1.5 μm each): NE: = 10,974; CATG = 9219. (A) Representative STED images of NE (top) and CATG (bottom) colabeled with nucleosomes. Scale bar: 2 μm; inset box: 2 μm × 2 μm. (B, C) Periodicity histograms for NE, CATG, and Nuc along NETs validate the pronounced periodicity of NE and Nuc (Figure 2). Averages of Gaussian fits are shown as colored lines. Mean ± SEM shown in black lines. (D) Centers of the Gaussian fits in (B) and (C), reflecting the predominant periodicity of each protein. Mean ± SEM. (E) Cross-correlation analysis of NE or CATG with nucleosomes, replotted from Figure S11. NE shows strong colocalization with nucleosomes, whereas CATG displays a multimodal distribution. Analysis includes only double-labeled NET fragments (NE: 16.2%; CATG: 1.5%). Median ± quartiles.

By using NanoNET for the cross-correlation analysis of STED images, we confirmed the strong colocalization of NE and PR3 with nucleosomes observed with SIM, while CATG showed no significant colocalization with nucleosomes (Figures 3E and S11). In summary, STED strengthens the finding that NE and PR3 share periodic binding sites with nucleosomes, while CATG follows a distinct binding mode without significant periodicity. Additionally, our results demonstrate that NanoNET can robustly analyze images acquired at different scales using independent super-resolution methods.

## Single-Molecule Localization Microscopy (SMLM) Reveals Subdiffraction Periodicity of NE and Nucleosomes on NETs

The periodicity of NET-associated proteins observed in SIM and STED and the colocalization with nucleosomes suggests that some NET proteins are highly organized along filaments and that this organization, in turn, may be afforded by the presence of a nucleosome. However, due to their limited resolution and sensitivity, both techniques might miss finer spatial (periodic) distributions. To overcome this limitation, we employed direct stochastic optical reconstruction microscopy (dSTORM),^21^ a single-molecule localization microscopy method that achieves a resolution of ∼20–30 nm on our SMLM system with single-molecule sensitivity.^22^

dSTORM experiments on NE and nucleosomes confirmed that both proteins form periodic clusters along individual NET filaments (Figure 4A). We quantified these periodic structures by generating periodicity histograms with the “NanoNET-Analysis” module on NET samples from multiple donors. In contrast to the single-Gaussian distribution of periodicities observed in SIM and STED, dSTORM revealed a bimodal distributed periodicity of both proteins, indicating two underlying distributions (Figure 4B). A two-component Gaussian fit of the data revealed two predominant periodicities, approximately ∼150 nm and ∼300 nm for both proteins, which represent integer multiples of each other (Figure 4C).

**Figure 4:**
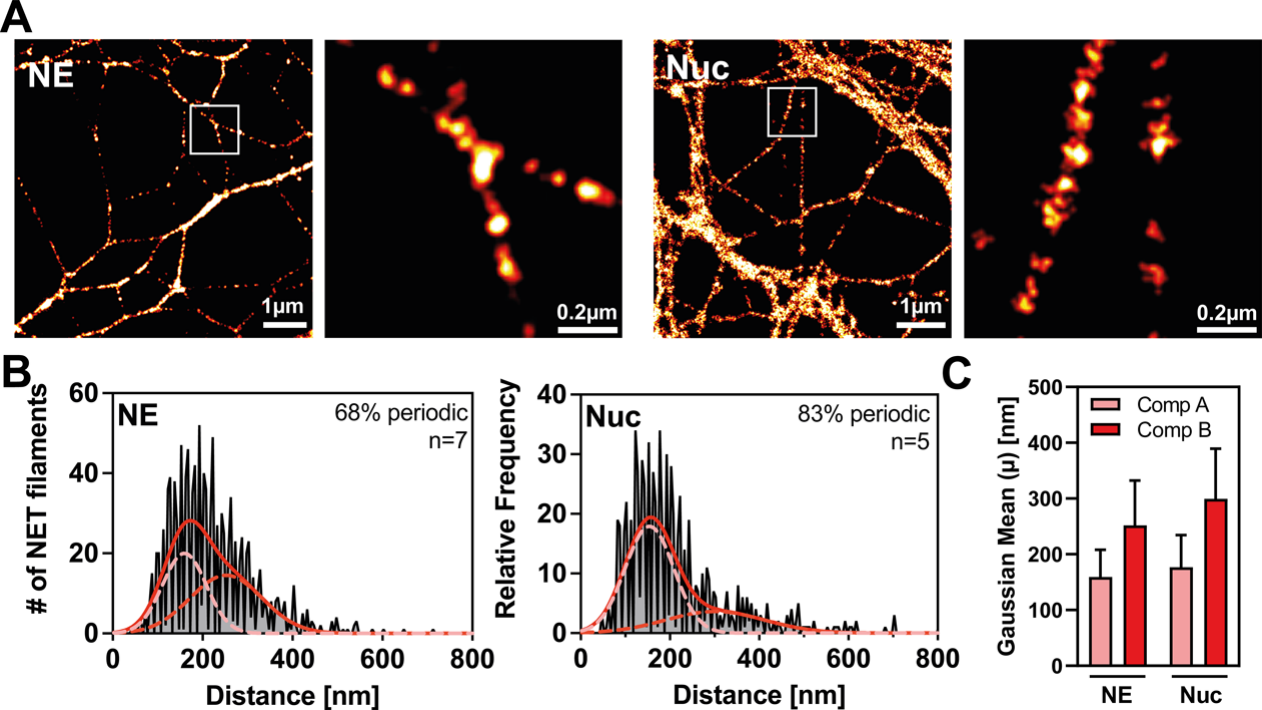
**SMLM reveals subdiffraction periodicity of NE and Nuc on NETs.** Colabeling of NE and nucleosomes (Nuc, mAB PL2.3) on NETs was acquired using SMLM (dSTORM). Data from seven (NE) and five (Nuc) independent donors; analyzed NET fragments: NE = 1084, Nuc = 773. (a) Representative images. NE and Nuc form clusters along single NET filaments. Scale bar = 1 μm (zoom 200 nm). (B) Periodicity histograms of the proteins displayed in (A). The exceptional resolution of SMLM reveals a bimodal periodicity (validated by the Akaike information criterion), in contrast to the single-Gaussian distribution observed in SIM/STED (Figures 2 and 3). Averages of the two-component Gaussian fits (solid red line) and individual components (dashed red lines) are displayed. (C) The two predominant periodicities (centers of both components from B) are plotted. Means ± SEM.

SIM and STED likely failed to detect the high-frequency component of the bimodal distribution due to their limited sensitivity and resolution, thereby missing periodic clusters with ∼150 nm spacing. The single-molecule sensitivity and higher resolution of SMLM resolved these missing structural repeats, offering a more detailed view of NET organization.

Taken together, this study provides an integrated workflow of sample preparation, super-resolution imaging, and bioimage analysis to investigate the nanoscale organization of NET-associated proteins. While SIM, STED, and SMLM are all powerful super-resolution methods that allow imaging with resolutions beyond the diffraction limit, each method has its own strengths and limitations regarding resolution, acquisition speed, and image processing. Here, we combined three complementary methods to show that a subset of NET proteins is organized into periodic patterns. To efficiently and robustly analyze super-resolution images of NET filaments for periodicity, we used NanoNET, our custom-built automated analysis toolbox.

Using NanoNET we identified and analyzed thousands of single DNA filaments for periodic binding patterns of NET proteins, thereby gaining novel insight into the nanoscale organization of NET associated proteins along single NET filaments. Our results demonstrate that a subset of NET binding proteins like NE and PR3 are arranged in a highly periodic fashion along NET filaments, mirroring the periodicity of nucleosomes on NETs. Moreover, both NE and PR3 are highly cross-correlated with nucleosomes, leading us to believe that these proteins likely depend either directly or indirectly on the positioning of nucleosomes on NETs to bind. Another NET binding protein, CATG, displays a more irregular pattern on NETs and weaker nucleosome correlation, despite its ∼90% structural similarity (∼30% sequence identity) to NE and PR3. The ∼20-fold stronger affinity of CATG for dsDNA than NE (*K*_d_ ≈ 35 nM vs 607 nM)^23^ supports a model in which CATG binds internucleosomal DNA directly, whereas NE and PR3 require additional interactions with histones or nucleosome-associated factors (Figure 5). Electrostatic interactions with the negatively charged DNA backbone alone cannot account for these differences, since NE, PR3, and CATG are all highly basic (pI 10.5, 9.4, and 8.9). Likewise, other basic serine proteases (e.g., trypsin: pI ∼9.3 and pancreatic elastase: pI ∼9.5–11) do not bind DNA.^24–26^ In addition, there may also be quaternary DNA structures that influence binding, that we could not account for.^24^

**Figure 5:**
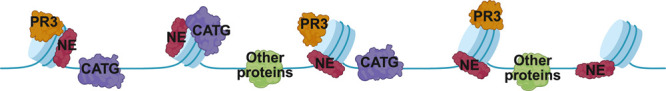
**Schematic binding model of different neutrophil serine proteases.** The positive colocalization of NE and PR3 with nucleosomes suggests that their binding to NETs is mediated predominantly through nucleosome association, in line with the shared periodic distribution. In contrast, CATG displays less pronounced periodicity and reduced colocalization with nucleosomes, indicating a mixed binding mode, potentially involving both nucleosomes and DNA or other components. All other NET-associated proteins tested lacked a periodic distribution and except for HNP-1 did not colocalize with nucleosomes, suggesting a nucleosome-independent binding mechanism. Together, this suggests that different neutrophil serine proteases use different modes of binding to NETs. This illustration was created with BioRender.

The average distance between nucleosomes reported within the nucleus is between 10 and 30 nm.^27,28^ Our super-resolution analyses show that in NETs this distance increases to ∼150 nm, suggesting that nucleosomes may be disassembled and evicted during NETosis. This apparent reduction in the total number of nucleosomes on NETs would be consistent with ATAC-see and ATAC-seq data acquired from resting neutrophil chromatin versus NETs showing large-scale changes in nucleosome occupancy and configuration on chromatin as NETosis proceeds.^29^ The eviction of nucleosomes from NETs might be functionally significant, allowing for decondensation of DNA as well as providing docking sites for proteins that decorate NETs independently of nucleosomes but dependently on DNA. As DNA binding has been implicated in modulating serine protease activity^23,30^ future work should determine whether the binding of NE, PR3, and CATG to DNA and/or nucleosomes influences their catalytic dependent- or independent functions in immunity. The periodic binding of proteins along NET filaments may also be functionally relevant. Regular spacing of highly charged proteins might generate electrostatic repulsion that contributes to filament integrity, thus preventing the condensation and collapse of individual NET filaments. On the other hand, ordered protein patterns might facilitate the cross-linking of NET filaments, contributing to the higher-order architecture of NETs. In addition, the periodic arrangement might also ensure the equal distribution of proteins across NETs that contributes to their microbicidal activity. Testing these possibilities will require sensitive, reproducible measurements along single filaments. NanoNET provides an automated framework for this quantification.

In summary, our nanoscale analyses show that some NET-associated proteins are specifically organized along individual NET filaments. Our newly designed open-access tools and protocols will enable researchers to scale up their investigations on how proteins organize along NET filaments. This nanoorganization of proteins within NETs will help generate new hypotheses with respect to NET formation and function. In addition to NETs, numerous other filamentous structures, including cytoskeletal elements or neuronal structures (as demonstrated in Figure S12), could be analyzed for periodic features using NanoNET. We regard NanoNET as a general tool that could be applied to study the distributions of fluorescent markers on filamentous structures at any scale.

## Supplementary Material



## Notes

Detailed experimental methods and materials; triple-color SIM of NET proteins (Supporting Figure S1); representative line profiles and autocorrelograms (Supporting Figure S2); SIM of PR3 and nucleosomes (Supporting Figure S3); colocalization analysis of NET proteins with nucleosomes (SIM) (Supporting Figure S4); dual-color controls (SIM) (Supporting Figure S5); PVL NET stimulation (SIM) (Supporting Figure S6); SIM of granular and cytosolic proteins (Supporting Figure S7); periodicity analysis of granular and cytosolic proteins (SIM) (Supporting Figure S8); multicolor STED microscopy of NETs (Supporting Figure S9); multicolor STED microscopy of PR3 and nucleosomes (Supporting Figure S10); colocalization analysis of NET proteins with nucleosomes (STED) (Supporting Figure S11); NanoNET is not limited to DNA NETs and reproduces known actin periodicity in neurons (Supporting Figure S12); primary antibodies (Supplementary Table 1); secondary antibodies (Supplementary Table 2); dyes and kits (Supplementary Table 3) (PDF)

## Abbreviations

SMLM: single-molecule localization microscopy
STORM: stochastic optical reconstruction microscopy
STED: stimulated emission depletion
SIM: structured illumination microscopy
SEM: scanning electron microscopy
NET: neutrophil extracellular trap
NSP: neutrophil serine protease
NE: neutrophil elastase
CATG: cathepsin G
PR3: proteinase 3
HNP-1: human neutrophil peptide 1
CAT: catalase
S100A9: calgranulin B
TALDO1: transaldolase
PMA: phorbol 12-myristate 13-acetate
PVL: Panton–Valentine leukocidin
CLSM: confocal laser scanning microscopy
