# Supplementary material for: Nanoscale Mapping Reveals Periodic Organization of Neutrophil Extracellular Trap Proteins
Source: Nano Lett. 2026 Apr 1;26(14):4564–71. doi: 10.1021/acs.nanolett.5c05175 (PMC13088356; doi:10.1021/acs.nanolett.5c05175)
Supplement: Supplementary file 1 [file nl5c05175_si_001.pdf]

SUPPORTING INFORMATION

Nanoscale Mapping Reveals Periodic Organization of Neutrophil Extracellular Trap Proteins

Moritz Winkler[1], Till G. A. Mack[3], Britta J. Eickholt[3], Jan Schmoranzer[2], Garth Lawrence Burn[1]*, Niclas Gimber[2]*

[1] Department of Cellular Microbiology, Max Planck Institute for Infection Biology, 10117 Berlin, Germany

[2] Advanced Medical Bioimaging Core Facility (AMBIO), Charité-Universitätsmedizin Berlin, 10117 Berlin, Germany

[3] Institute of Biochemistry and Molecular Biology, Charité-Universitätsmedizin Berlin, 10117 Berlin, Germany

* Correspondence should be addressed to Niclas Gimber (niclas.gimber@charite.de) and Garth Burn (burn@mpiib-berlin.mpg.de).

SUPPORTING FIGURES

Supporting Figure S1. Triple-color SIM of NET proteins.

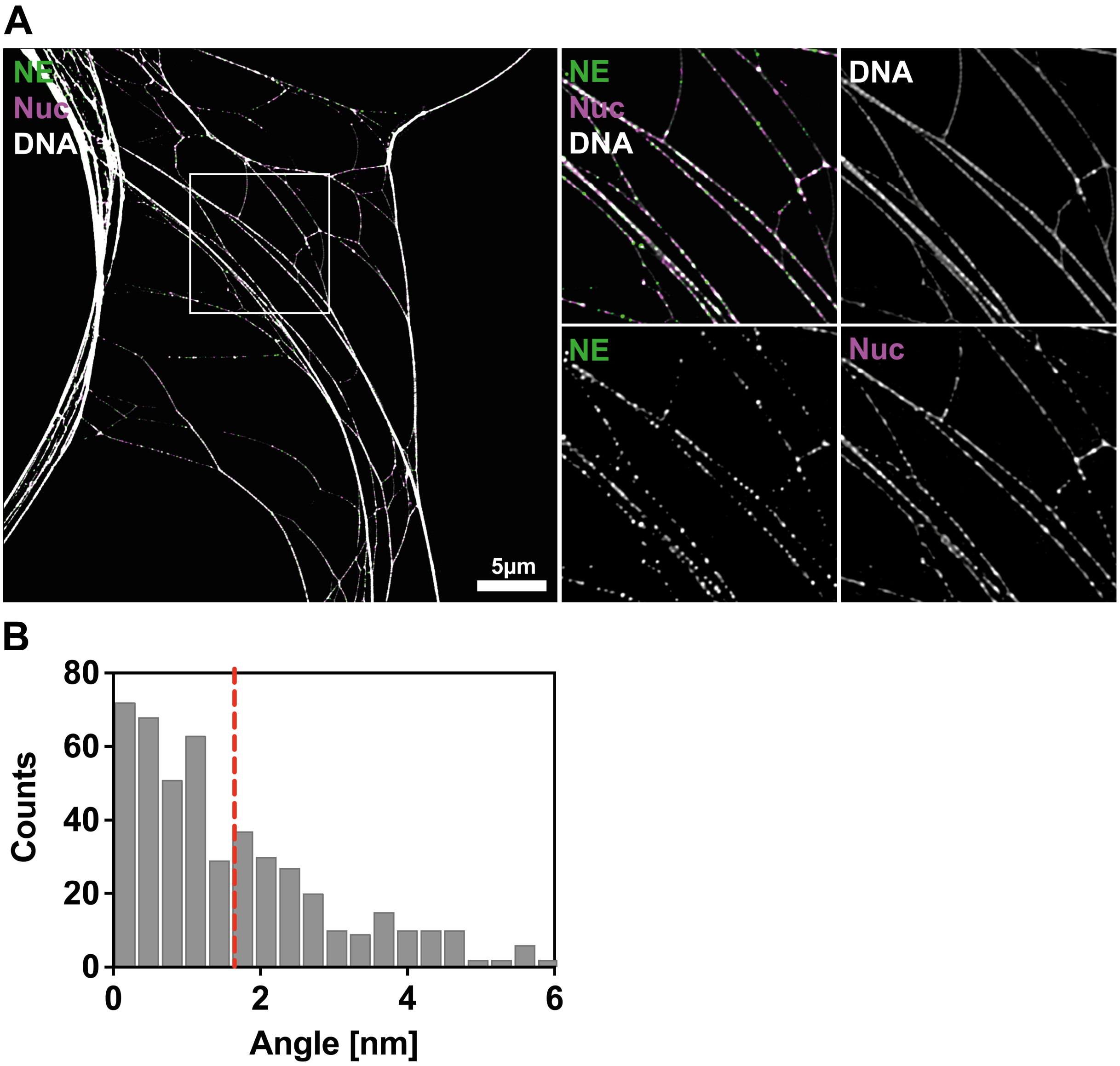

Supporting Figure S1: Triple-color SIM of NET proteins. (a) The DNA backbone (white) was stained with the intercalating dye YOYO-1 and co-labeled with antibodies against NE (green) and Nuc (purple). The overview image (left) reveals the characteristic web-like structure of NETs, including individual filaments and unresolved dense regions, which were excluded from analysis. Enlarged regions (right, 10 × 10 µm) highlight the uniformly labeled DNA backbone, while NE and Nuc appear in periodic clusters along the filaments. **(b)** Quantification of sample flatness: Quantification of the local axial slope between neighboring nucleosome clusters in (a). The median axial slope of 1.6° (dashed line) leads to only a negligible underestimation of true inter-cluster distances (below 0.04%) after maximum intensity projection of the 3D stack into two dimensions.

Supporting Figure S2. Representative line profiles and autocorrelograms.

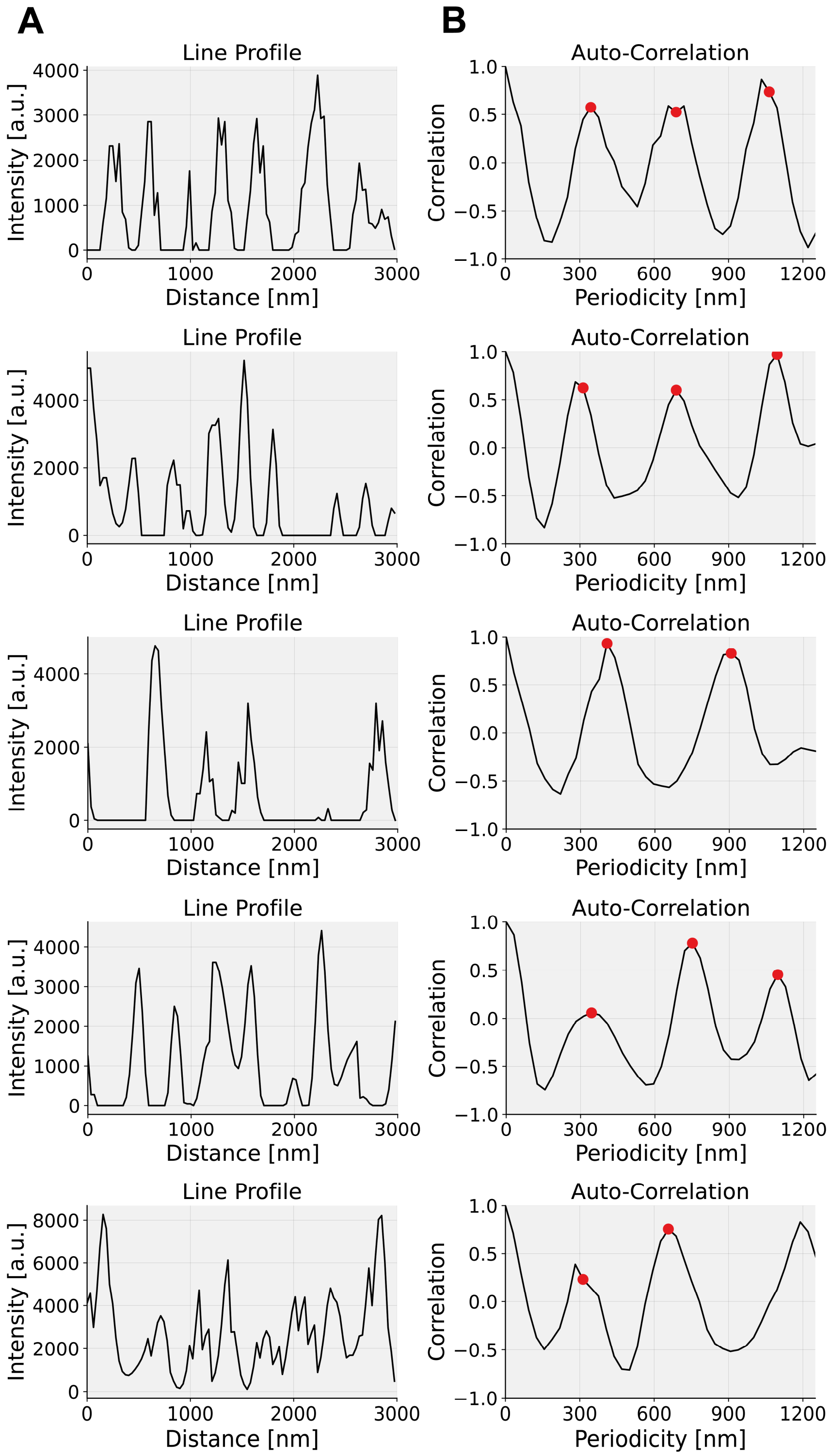

Supporting Figure S2. Representative line profiles and autocorrelograms. (a) NanoNET-derived line intensity profiles of nucleosome signal along NETs (SIM data). **(b)** Corresponding autocorrelograms of the line profiles shown in (a). Red dots mark local maxima (dominant periodicities) automatically detected by NanoNET. The first non-zero peak was used to generate periodicity histograms.

Supporting Figure S3. SIM of PR3 and nucleosomes.

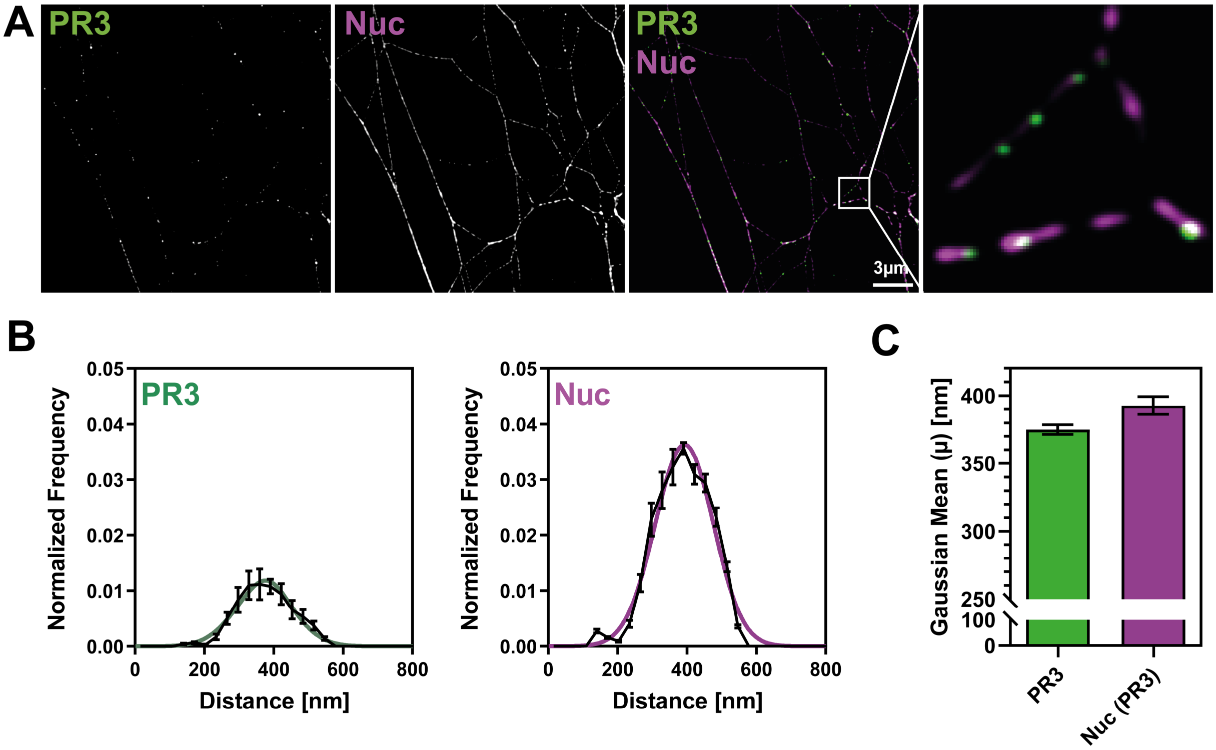

Supporting Figure S3: SIM of PR3 and nucleosomes on NETs. Co-labeling of PR3 and NUC (mAB PL2.3) on NETs. **(a)** Representative images. Scale bar = 3 µm, box = 2 × 2 µm. **(b)** Periodicity histograms of the proteins displayed in (a) with average of Gaussian fits (colored lines). Data from 5 independent donors; analyzed NET fragments (1.5 µm each): 25,830. Both PR3 and Nuc show strong periodicity, as indicated by the high peak of the periodicity histograms. Means ± SEM (black lines). Centers from Gaussian fits (b) were plotted in **(c)** and reveal similar gaussian distributions for PR3 and Nuc. Means ± SEM.

Supporting Figure S4. Colocalization analysis of NET proteins with nucleosomes (SIM).

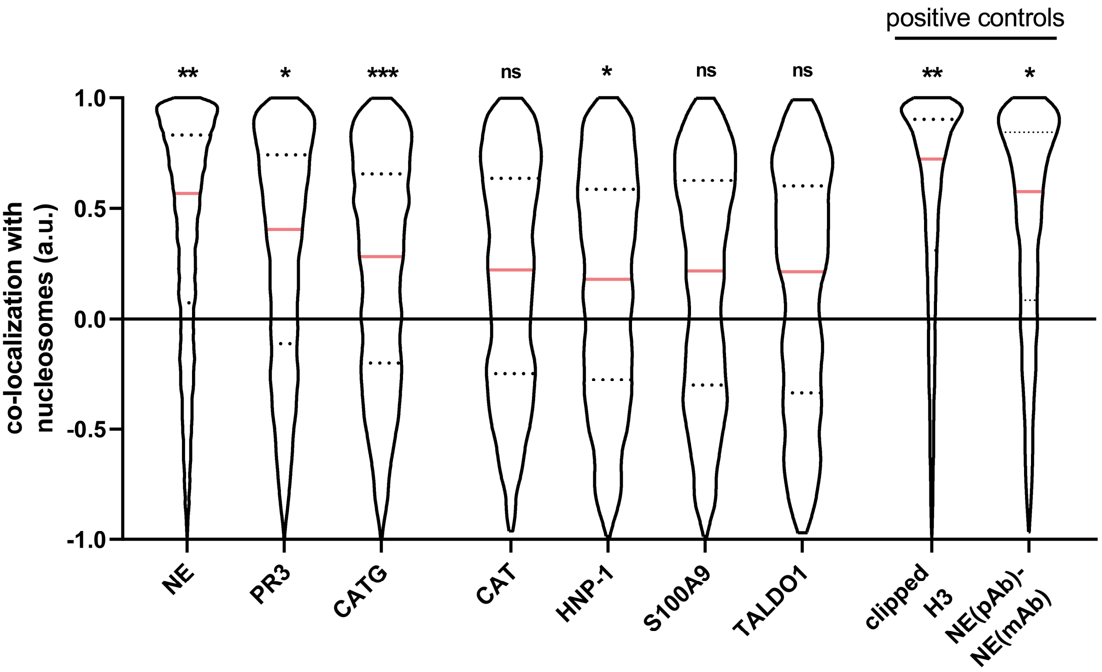

Supporting Figure S4: Colocalization analysis of NET proteins with nucleosomes (SIM). NET-associated proteins with nucleosomes and technical positive controls were analyzed. NE, PR3, CATG and HNP-1 and clipped Histone 3 (biological positive control, as in [1]) show significant colocalization with nucleosomes. While NE and PR3 co-localize with nucleosomes to a similar extent as the positive controls (clipped H3 / nucleosomaes and pAB NE / mAB NE), CATG and HNP-1 display only weak colocalization. CAT, S100A9, and TALDO1 exhibit multimodal distributions and no significant colocalization. Median ± quartiles. Data from 3-5 independent donors. Analyzed line profiles and fraction of double-labeled (1.5 µm each): NE = 19,691 (32.6%), PR3 = 6,847 (26.5%), CATG = 7,442 (11.2%), CAT = 2,215 (3.8%), HNP-1 = 1,903 (2.7%), S100A9 = 2,000 (5.9%), TALDO1 = 1,042 (1.2%), clipped H3 = 13,128 (24%), NE (pAb) – NE (mAb) = 2,553 (17.6%). P-values (one-sample t-test against zero, pooled per donor, Bonferroni-corrected): NE: 0.004, PR3: 0.041, CATG: 0.001, CAT: 0.063, HNP-1: 0.004, S100A9: 0.096, TALDO1: 0.081, clipped H3: 0.004, NE (pAb) – NE (mAb): 0.019. * $p < 0.05$, ** $p < 0.01$, *** $p < 0.001$, n.s. not significant.

Supporting Figure S5. Dual-color controls (SIM).

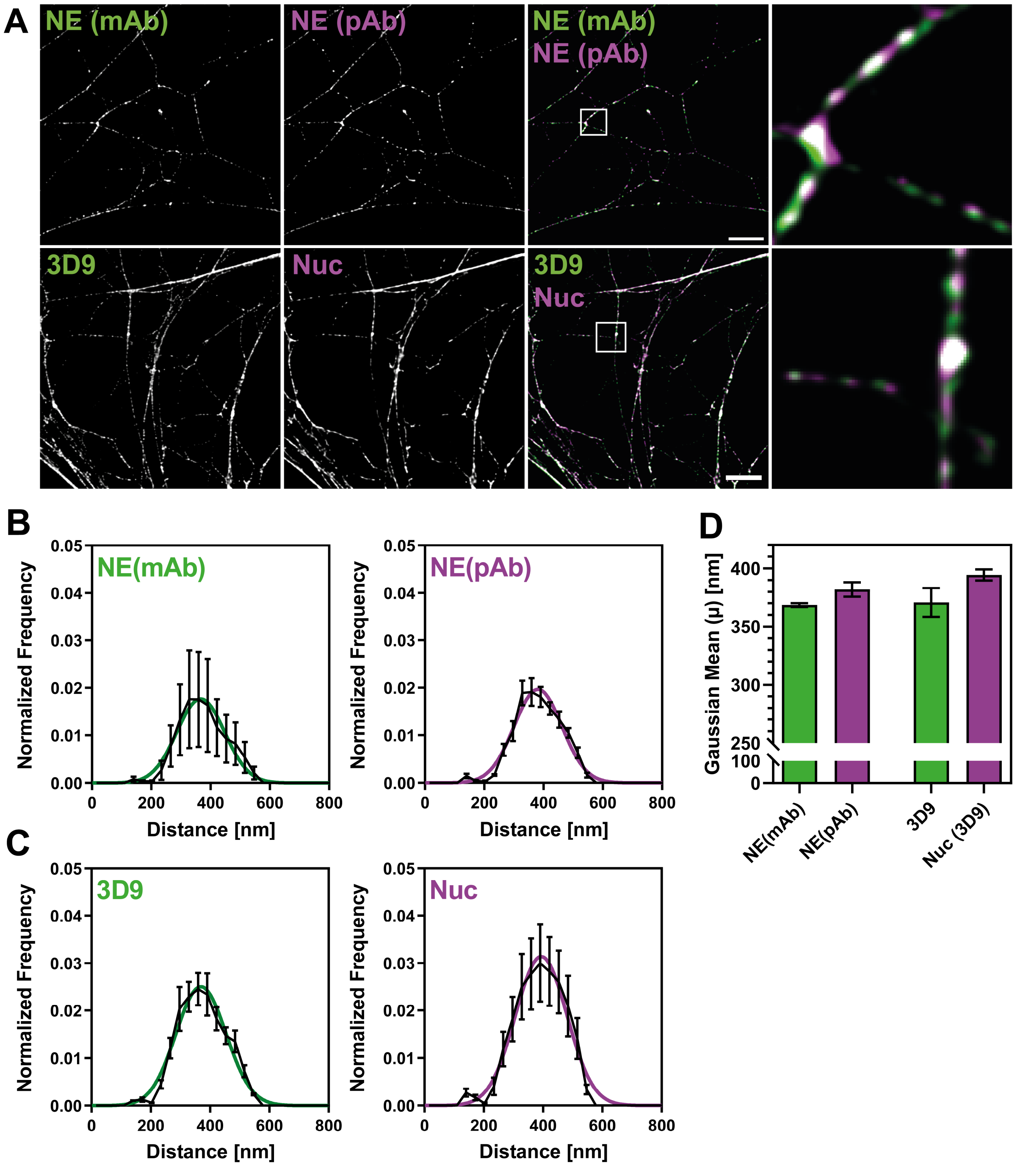

Supporting Figure S5: Dual-color controls (SIM). (a) Co-labeling of NE using polyclonal (pAb) and monoclonal (mAb) antibodies (top row), and nucleosomes using the cleaved histone H3 antibody (3D9) and the nucleosome marker (mAB PL2.3) (bottom row). Both NE antibodies co-localize. The same is true for both nucleosomal markers. Analyzed fragments (1.5 µm each): NE (mAb) - NE (pAb) = 14466 from 3 independent donors; 3D9 - Nuc = 54712 from 4 independent donors. Scale bar = 3 µm, boxes = 2 × 2 µm. **(b,c)** Periodicity histogram of the proteins displayed in (a) with average of Gaussian fits (colored lines). Centers from Gaussian fits (b,c) were plotted in **(d)** and served as a measure for the predominant periodicity. Means ± SEM.

Supporting Figure S6. PVL NET stimulation (SIM).

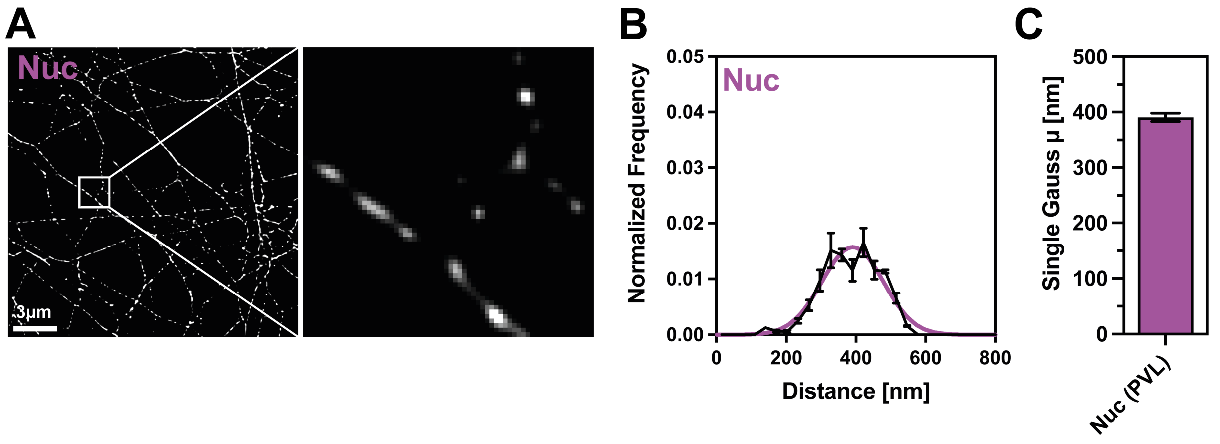

Supporting Figure S6: **PVL NET stimulation (SIM). (a)** Labeling of nucleosomes (mAB PL2.3). Neutrophils were (in contrast to all other experiments) stimulated with Panton Valentine Leukotoxin (PVL; a pore-forming toxin produced by Staphylococcus aureus) that, in human neutrophils, induces NETosis[2]. Analyzed NET fragments (1.5 µm each): 23634 from 2 independent donors **(b)** Periodicity histograms for (a) along NETs. The Gaussian fit is shown as colored line. The center of the Gaussian fit (b) was plotted in **(c)** and served as a measure for the predominant periodicity. Means ± SEM. Note the similar periodicity after this NADPH oxidase-independent stimulus and after the NADPH oxidase-dependent stimulus (PMA, Figure S5).

Supporting Figure S7. SIM of granular and cytosolic proteins.

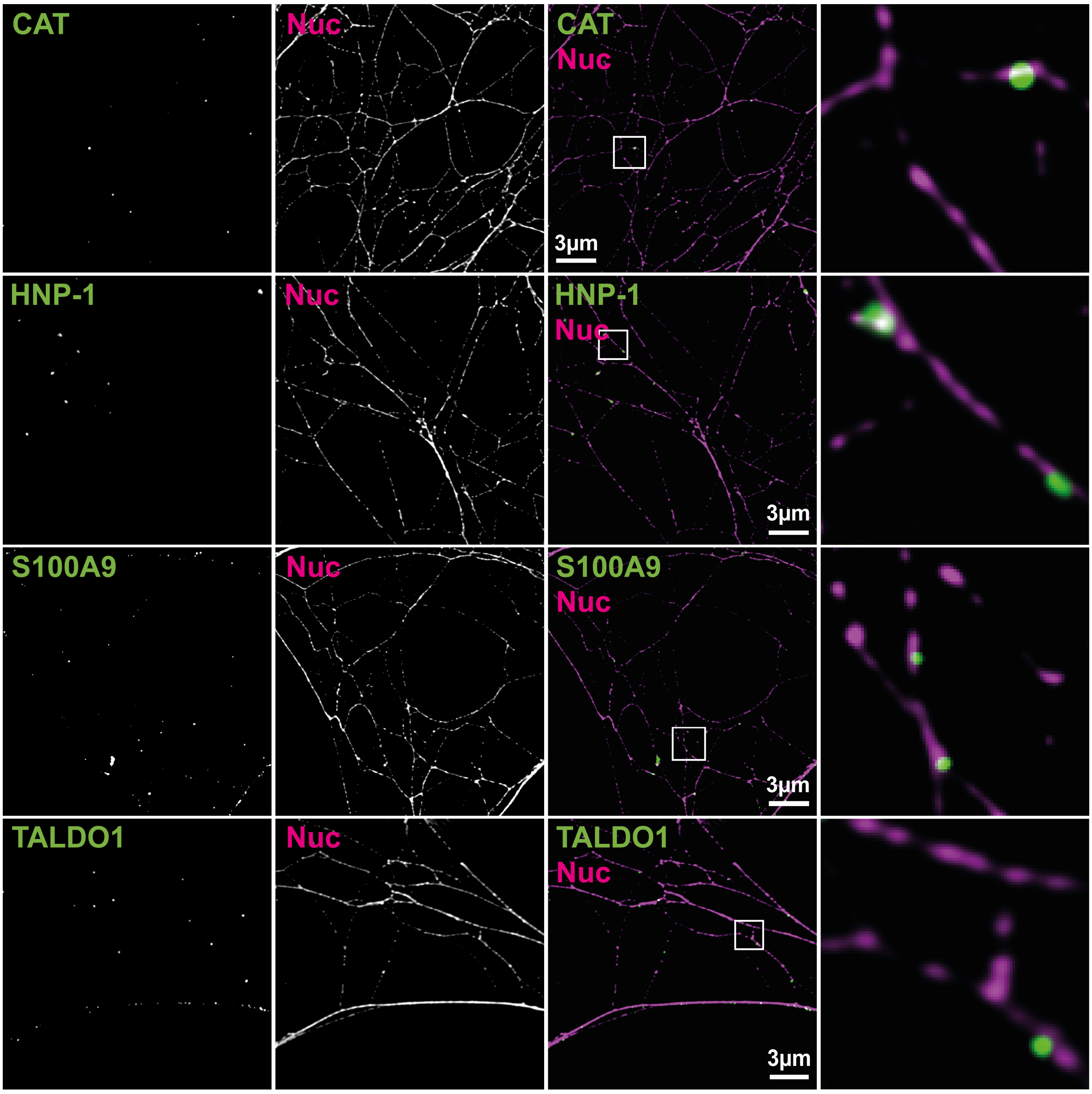

Supporting Figure S7: SIM of granular and cytosolic proteins. Co-immunolabeling of granular proteins CAT and HNP-1, and cytosolic proteins S100A9 and TALDO1 with nucleosomes (Nuc, magenta; mAb PL2.3) on NETs. All proteins show low-abundant labeling and no obvious periodicity. HNP-1 and S100A9 occasionally form large clusters that co-localize with nucleosomes. Overall, colocalization with nucleosomes is low. Scale bar = 3 µm, boxes = 2 × 2 µm.

Supporting Figure S8. Periodicity analysis of granular and cytosolic proteins (SIM).

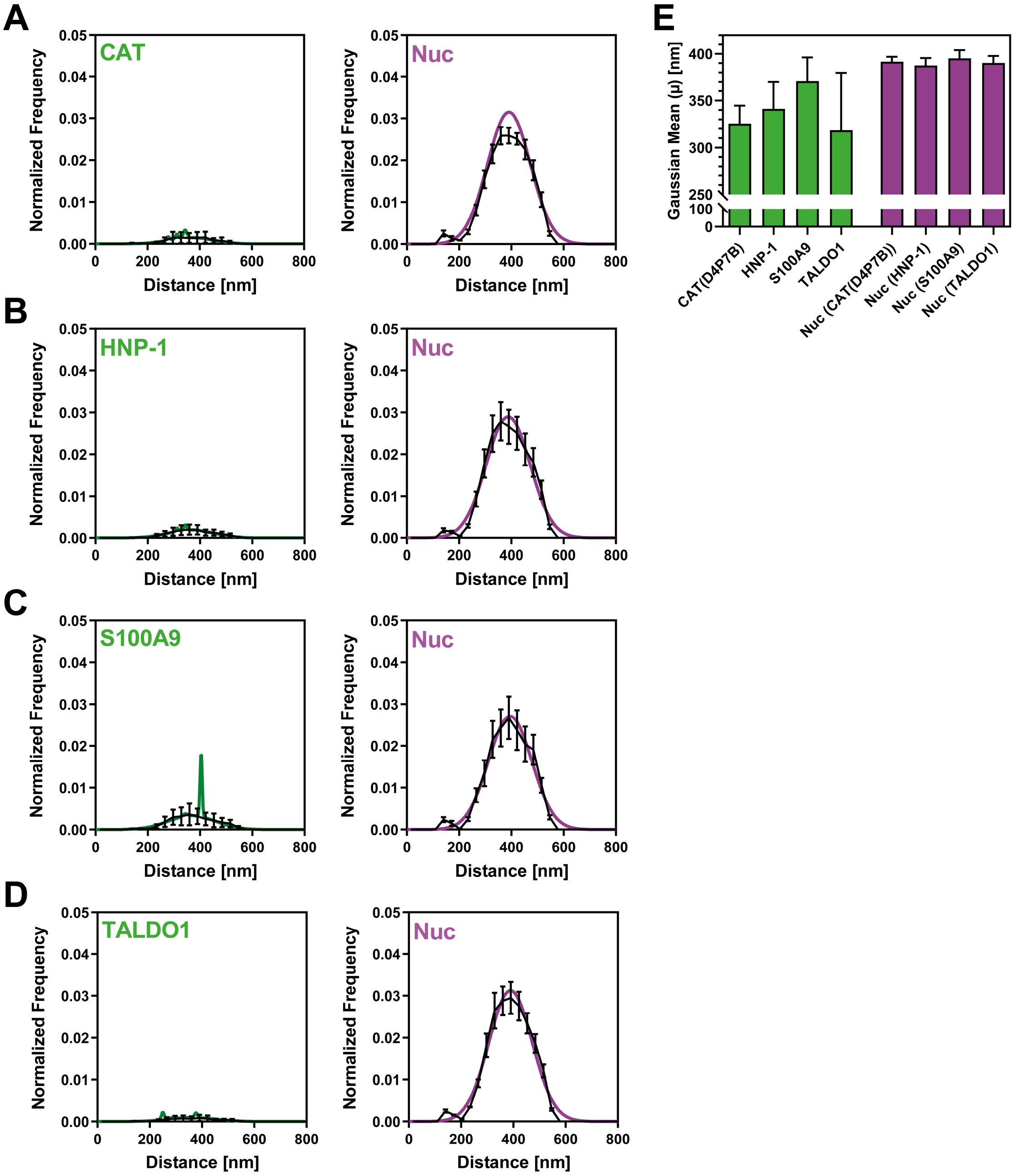

Supporting Figure S8: Periodicity analysis of granular and cytosolic proteins (SIM). (a-d) Periodicity histograms of granular (HNP-1, CAT) and cytosolic (S100A9, TALDO1) neutrophil proteins shown in Sup Figure 7, with averages of Gaussian fits (colored lines). All proteins exhibit low periodicity, as indicated by weak peak in the periodicity histogram. Data from four independent donors; analyzed NET fragments (1.5 µm each): HNP-1 = 70,367; S100A9 = 33,890; CAT = 58,019; TALDO1 = 85,005. Means ± SEM (black lines). **(e)** Centers from Gaussian fits (b,c) were plotted in (a-d) and serve as a measure for the predominant periodicity. Means ± SEM.

Supporting Figure S9: Multi-color STED microscopy of NETs.

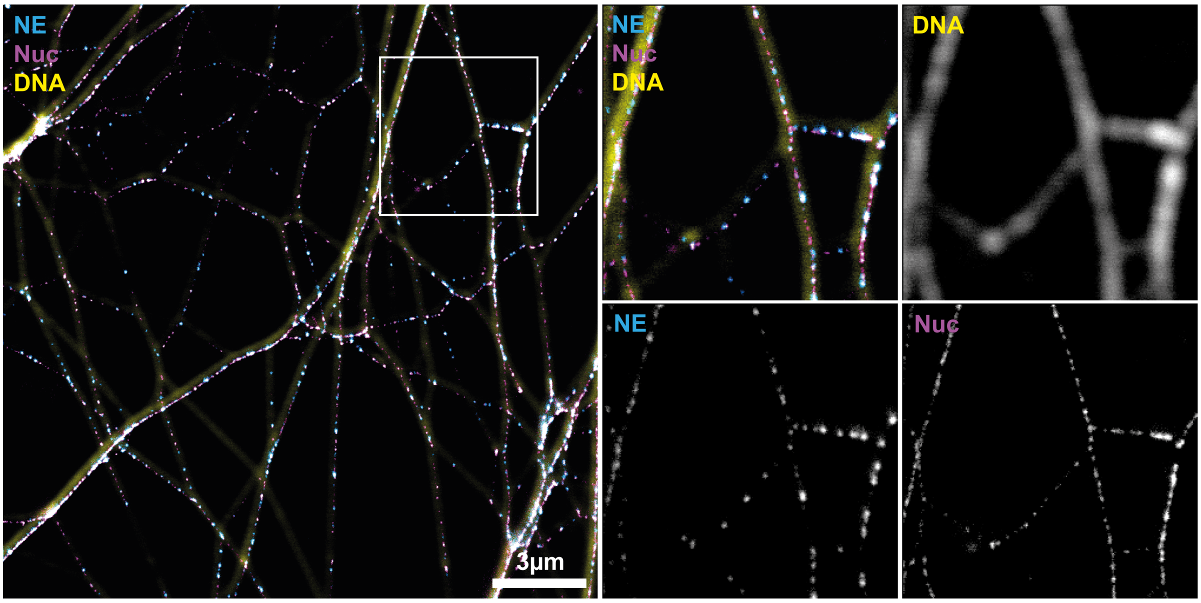

Supporting Figure S9: Multi-color STED microscopy of NETs.
The DNA backbone (yellow) was stained with the DNA-intercalating dye YOYO-1 and imaged in confocal mode. NETs were co-labeled with antibodies against NE (cyan) and Nuc (magenta) and imaged using STED. The overview image (left) shows web-like structure of NETs with individual filaments. NE and Nuc are organized in periodic clusters along the filaments (zoom, right). Diffraction limited confocal images of the DNA channel reveal continues labelling on NET filaments, which was used for NET segmentation. Zoom: 5 × 5 µm.

Supporting Figure S10. Multi-color STED microscopy of PR3 and nucleosomes.

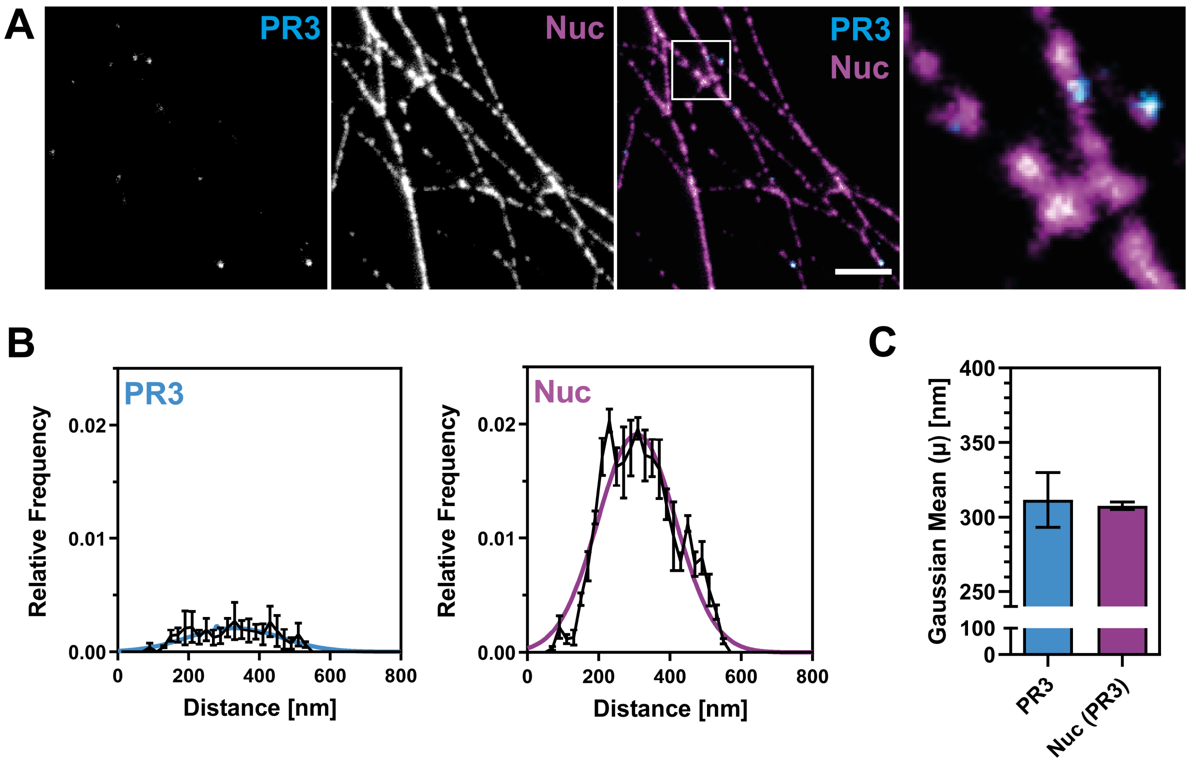

Supporting Figure S10: Multi-color STED microscopy of PR3 and nucleosomes. (a) Co-labeling of PR3 and nucleosomes (Nuc) on NETs in STED mode. Scale bar = 2 µm, boxes = 2 × 2 µm. **(b)** Periodicity histogram of the proteins displayed in (a) with average Gaussian fits (colored lines). Both PR3 and Nuc display a clear peak in the periodicity histogram. Data from three independent donors; analyzed NET fragments: NE = 10,974, CATG = 9,219, PR3 = 7,460. Means ± SEM (black lines). Peaks from the Gaussian fits (b) were plotted in **(c)** and serve as a measure of the predominant periodicity. Means ± SEM.

Supporting Figure S11. Colocalization analysis of NET proteins with nucleosomes (STED).

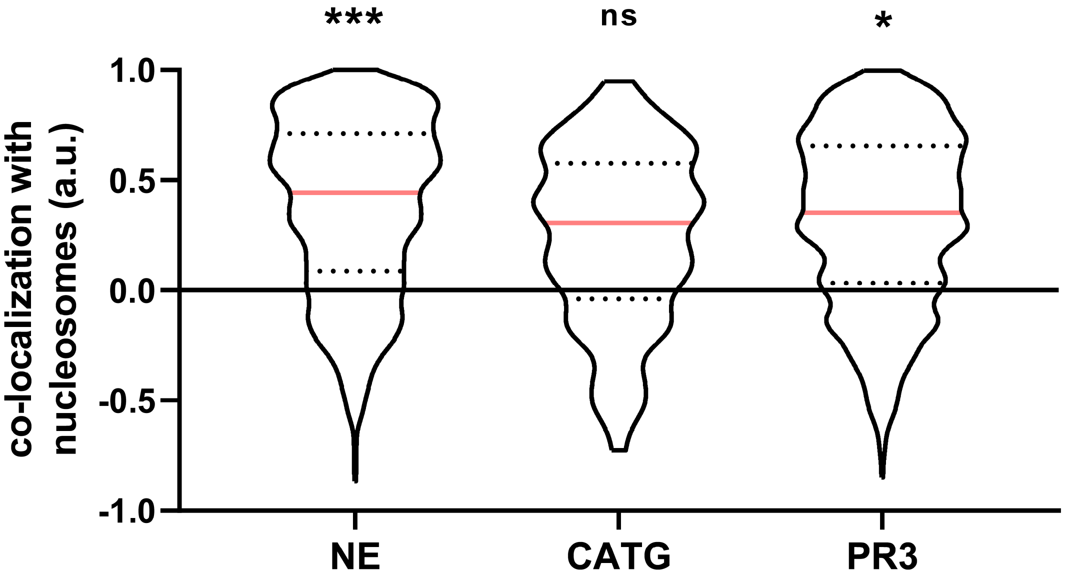

Supporting Figure S11: Colocalization analysis of NET proteins with nucleosomes (STED). NE and PR3 show significant colocalization with nucleosomes, while CATG (in contrast to SIM, Figure 4) shows no significant colocalization. Median ± quartiles. Data from three independent donors. Analyzed line profiles and fraction of double-labeled (1.5 µm each): NE = 10,974 (16%), PR3 = 7,460 (7%), CATG = 9,219 (1%). P-values (one-sample t-test against zero, pooled per donor, Bonferroni-corrected): NE: 0.0004%, PR3: 0.043, CATG: 0.056. * $p < 0.05$, *** $p < 0.001$, n.s. not significant.

Supporting Figure S12. NanoNET is not limited to DNA NETs and reproduces known actin periodicity in neurons.

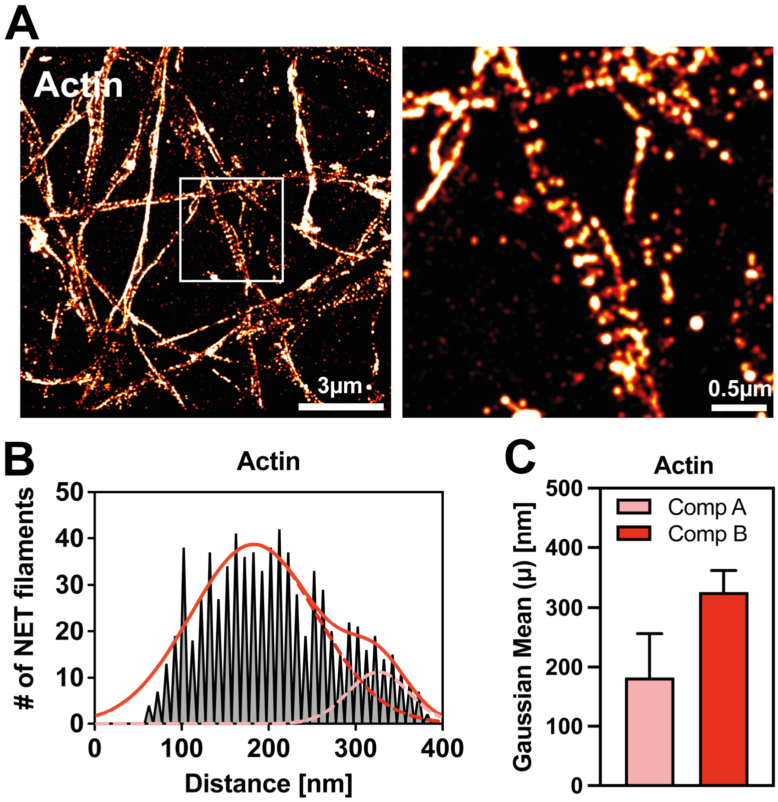

Supporting Figure S12: **NanoNET is not limited to DNA NETs and reproduces known actin periodicity in neurons.** Actin labeling of hippocampal neurons was imaged by SMLM (dSTORM) and analyzed for spatial periodicities using the NanoNET toolbox. Analyzed NET fragments: 1026 from 11 images. **(a)** Representative SMLM image showing actin organized into periodic structures along neuronal processes. **(b)** Periodicity histogram of actin localizations from the region shown in (a) revealing a bi-modal periodicity, validated by Akaike information criterion. The two-component Gaussian fit (solid red line) and individual Gaussian components (dashed red lines) are shown. **(c)** Quantification of the two predominant periodicities (centers of the Gaussian components in b). Mean ± SEM. Note the high-frequency component (183 nm) precisely matches the previously reported axonal actin ring spacing measured by SMLM (182 nm [3]) while the second component approximates twice this spacing. Together, these results demonstrate that NanoNET reliably reproduces known sub-diffraction periodicities and can be applied to filamentous structures beyond NETs.

METHODS

Human sample collection and cell lines

Our study was conducted in accordance with the Helsinki Declaration. Healthy blood samples were collected according to the approval and guidelines of the local ethics committee (EA1/0104/06).

Neutrophil isolation and cell culture

Blood was collected into EDTA containing tubes, layered 1:1 on Histopaque 1119 (Sigma) followed by centrifugation for 20 min at 800 × g. Plasma and the upper layers of the separated blood, consisting mainly of peripheral blood mononuclear cells, were discarded. The neutrophil-rich pink layer was collected whilst the densest layer consisting of red blood cells was left undisturbed. Neutrophils were washed in PBS containing 0.1% human serum albumin (HSA, Grifols), and further fractionated on a discontinuous Percoll (Pharmacia) gradient consisting of 2mL layers with densities of 1105 g/ml (85%), 1100 g/ml (80%), 1093 g/ml (75%), 1087 g/ml (70%), and 1081 g/ml (65%). Neutrophils were carefully layered on the top of the gradient and centrifuged for 20 min at 800 × g. The interface between the 80% and 85% Percoll layers was collected and washed with PBS containing 0.05% HSA. Neutrophil purity was determined to be > 95% by flow cytometry.

NET preparation for super resolution microscopy

Isolated neutrophils were seeded on isopropanol sonicated high-precision coverslips (⌀24 mm, 1.5H, Marienfeld, Germany) in 6-well cell culture dishes at a density of $1.5x10^5$ cells/coverslip in 2ml RPMI medium + 0.1% HSA. Cells were allowed to adhere to the coverslip for 15min at 37°C, 5% CO_2. NET formation was stimulated by incubation with 100 nM PMA (Sigma) for 3:30h at 37°C, 5% CO_2. Alternatively (if indicated), equal volumes of PVL toxin components LukS and LukF (IBT Bioservices) were mixed, and neutrophils were stimulated with 10nM of PVL toxin mixture for 4h at 37°C, 5% CO_2.

Throughout the NET preparation, extra attention was used not to shake or disturb the solution to preserve the delicate structure of NETs. NET formation was validated by light microscopy. Samples were fixed in 3% para-formaldehyde (PFA) (w/v) (Electron Microscopy Sciences) for 12 min, RT by adding 1ml of 3x concentrated PFA to each coverslip within the 6-well cell culture dish. Coverslips were subsequently washed twice with PBS. For immunofluorescent staining, the samples were permeabilized for 10 min with 1% Triton X-100 in PBS at RT and

subsequently blocked in fish gelatin/goat serum blocking buffer for 1h, RT. Afterwards, samples were incubated with primary antibodies in fish gelatin/goat serum blocking buffer (0.05% (v/v) Tween 20, 3% (v/v) normal goat serum, 3% (w/v) freshwater fish gelatin, 1% (w/v) BSA in PBS pH 7.5) overnight at 4°C. After two subsequent washes with PBS, secondary antibodies and DNA dyes were added to coverslips in fish gelatin/goat serum blocking buffer for 1h at RT. Where required, samples were sequentially labeled with primary antibodies from the same host species. Primary antibodies were conjugated to fluorophores by NHS ester labeling according to manufactures protocol (Thermo, # A88068) and added to samples for 1h at room temperature. Samples for STORM were mounted on concave microscopy slides with 100µl oxygen scavenging buffer (0.1 mg/ml GLOX, 0.1 mg/ml HRP, 25mM HEPES, 5% glycerol, 25mM glucose in PBS, pH 6.0) and sealed with a two-component dental imprint material to enable effective photo switching of fluorescent cyanine dyes. Preparation of buffers and mounting of samples was performed immediately before image acquisition. For SIM and STED, coverslips were mounted to HistoBond microscopy slides (Marienfeld, Germany) in three drops ProLonged Glass antifade mountant (ne=1.52, Invitrogen). Mounted NET samples were cured for at least one week at RT before imaging to minimize DNA filament snapping at lower wavelength laser powers.

Neuron preparation for super resolution microscopy

The animal study (Figure S12) was approved by Landesamt für Gesundheit und Soziales (LaGeSo; Regional Office for Health and Social Affairs) in Berlin. The study was conducted in accordance with the local legislation and institutional requirements under permit number T-CH0025/23.

Mouse hippocampal neuron cultures were prepared as described previously[4]. Neurons were maintained for 21 days in vitro (DIV21), fixed, and processed for F-actin labeling according to the published protocol. Briefly, cells were permeabilized for 10 min in PHEM buffer (PIPES, HEPES, EDTA, and $MgCl_2$; pH 7.4) containing 0.1% Triton X-100, then blocked in PHEM supplemented with 4% goat serum. F-actin was labeled with a mixture of phalloidin-conjugated fluorophores (Alexa Fluor 647 and Atto 488; final concentrations ~264 nM and 20 nM, respectively). Suitable regions of interest were identified, and focus was adjusted using epifluorescence illumination in the 488nm channel. dSTORM images were then acquired in the 647nm channel as described below.

SIM microscope setup, image acquisition and reconstruction

Structured Illumination Microscopy (SIM) was performed using a Zeiss ELYRA 7 Lattice SIM system on an Axio Observer 7 stand. The microscopy was equipped with a Zeiss Plan-Apochromat 63x/1.4 oil DIC M27 objective and HR diode 488nm (500mW), HR DPSS 561nm (500mW) and HR diode 642nm (500mW) laser lines. Simultaneous detection of two channels was achieved by Duolink adaptor, operating two pco.edge 4.2 CL HS sCMOS cameras (6.5µm pixel size, peak QE 82%, liquid cooled) (PCO AG, Kelheim, Germany). Excitation and emission beams were split using the DuoLink SR QUAD filter module with double emission bands and a dichroic mirror (SBS LP 560 with EF BP420-480/BP495-550 & EF570-620 & LP655). Combined with acousto-optic tunable filter (AOTF) to switch laser lines, this setup enabled quick four-channel image acquisition with the blue and orange as well as the green and red spectra in parallel. Prior to sample imaging, the sCMOS cameras were aligned using the in-built alignment option of the ZEN Black software. Samples were loaded to the microscopy using Immersol 518 F / 30°C (ne=1.518, Zeiss) to match the refractive index of both glass and ProLong Glass (Invitrogen) mountant. Acquisitions were performed with 50-100mW laser powers at 10-30ms exposure for antibody labelling (568nm, 647nm lasers) and 150-200mW laser power at 50ms exposure for YOYO-1 (Invitrogen) DNA labeling (488nm laser), respectively. For image acquisition, 15-20 regions of interest (2048x2048px) were selected per sample and 3.3µm z-stack (30 slices, 0.11µm intervals) were acquired in optimal mode, switching tracks in "frame fast" mode. Structured illumination was set to 'Lattice SIM' with a grating of 13 phases. Channel alignment was performed individually for every session using a sample control. NETs were stained for nucleosomes (anti-PL2.3) using secondary antibodies in all channels (Alexa488, Alexa568, STAR635P), and the channel alignment tool (ZEN black software) was used in 'affine mode' using markers to generate an alignment matrix. Afterwards, images were reconstructed using the Zeiss SIM2 module in 'strong fixed' mode including the channel alignment matrix. Zeiss SIM² is a proprietary algorithm increasing signal-to-noise ratio, minimizing SIM artifacts. The quality of the SIM reconstructions were validated using SIMcheck[5] all diagnostics passed the test. In particular, the inspection of the Fourier space of the reconstructed images revealed no discrete peaks or artefacts, confirming that the observed periodicities reflected the biological nano-structures.

STED microscope setup, image acquisition and reconstruction

Stimulated emission depletion (STED) microscopy was performed on an Abberior STED Facility Line (#FC211501, Abberior GmbH, Göttingen) based on Olympus IX83 Inverted Microscope. The microscope was equipped with a UPXLAPO apochromat 60x/1.42 NA oil objective (Olympus Life Sciences), 405nm/cw (50mW), 485nm/pulsed (~1mW, 40MHz), 561nm/pulsed (~200µW, 40MHz), 640nm/pulsed (~1mW, 40MHz) excitation laser lines and ultra-high power 775nm/pulsed (>2750mW, 40MHz, repetition rate 25-40MHz) STED laser. The laserlines of the microscope were aligned using the Abberior Nanoparticle Autoalignment Slide (NP-3016) and the inbuilt auto-alignment tool in the Inspector software. STED images were acquired in 15x15µm fields-of-view, with a resolution of 20nm/px. NET proteins were imaged using appropriate antibodies and a STAR Orange conjugated secondary antibody by CLSM (30% laser power; 15-22ms) and STED (45% laser power; 30-40ms) with the 561nm laser. Nucleosomes were imaged using the PL2.3 antibody and a STAR 635P conjugated secondary antibody by CLSM (1.5% laser power, 8-15ms) and STED (2.5% laser power, 12-24ms) with the 640nm laser. In STED mode, the 775nm depletion laser was used at 30% laser power and 10ms exposure.

STORM microscope setup, acquisition reconstruction and line profile generation

Single molecule localization microscopy (dSTORM) was performed at the Advanced Medical Bio Imaging (AMBIO) facility in the Charité Universitätsmedizin campus Mitte. A Nikon Ti Eclipse based STORM microscope system (N-STORM V3) was used[6]. The EPI-Total Internal Reflection Fluorescence (TIRF) microscope was equipped with an Agilent MLC400 laser box (405nm, 488nm, 561nm, 640nm), a high NA oil objective (100x 1,49N.A.) and a sCMOS camera (Prime 95B, 1024x1024, Photometrics). Optical Filters were from AHF Analysentechnik. The microscope and camera were controlled by NIS Elements software (Nikon) and image analysis was performed in Fiji (ImageJ 1.54c). The dSTORM images were acquired using a highly inclined and laminated optical sheet (HILO) to minimize background staining and facilitated the imaging of thin DNA filaments. Images were acquired at a frame rate of 50 Hz (20 ms) for AlexaFluor 647 labeled samples with a total of 15,000 frames. Single molecules were localized with the Fiji plugin ThunderSTORM [7] using a pixel size of 110nm, 2.5 photoelectrons per A/D count and a base level of 100 photons. Image de-noising was performed using a third order wavelet filter (B-Spline) and molecules were localized using the local maximum method [2*std(Wave.F1)] as a peak intensity threshold. Sub pixel localization was performed using the integrated Gaussian point-spread function (PSF) with a fitting radius of 7 pixel. After the average shifted histogram reconstruction, data was density filtered (5 neighbors, 50nm radius) and drift corrected. The results were exported as localization lists and reconstructed as images (average shifted histogram). Since the single-color dSTORM images lacked DNA labeling required for automated NET detection, filament line profiles were manually selected using ImageJ. The "free-hand selection" tool with a 5-pixel width was employed to choose ROIs representing single NET filaments. These were defined by localizations with a minimal width and by exclusion of filament branches or links. Moreover, to mitigate potential errors arising from data nature or missing localizations, a maximum profile length of 1-2 µm was set. This is in line with previously described autocorrelations for filament analysis[8]. Selected areas were saved as ROIs and line profiles were exported as .csv files.

Generation of NET filament line profiles

The z-range encompassing the NET signal was manually selected from the images, followed by the generation of maximum projections of 3d stacks (SIM and STED) from selected areas. Background values were determined from NET-free areas and subtracted from all images per NET preparation before further processing. Images were excluded if (i) a substantial fraction of the field of view contained clumped/collapsed NETs, (ii) the DNA signal was too weak to reliably segment the filament backbone, (iii) the NET protein labelling was too weak to follow filaments, and/or (iv) obvious imaging artefacts were present (e.g., strong background or inhomogeneous illumination). Applying these criteria resulted in the exclusion of ~30% of acquired images, all remaining images were included in the analysis. Additionally, non-NET regions, such as remnants of neutrophils, were excluded through a mask to ensure that subsequent analyses focused exclusively on the NET structures. While regions in dSTORM images were manually selected, the analysis workflow **"NET-Detection"** from our custom-built **NanoNET toolbox** (https://github.com/ngimber/NanoNET) was employed for automated NET segmentation in SIM and STED images. The NET-detection workflow of NanoNET features a graphical user interface (GUI, Figure 1) to adapt critical parameters. It employs a series of filters to generate line intensity profiles. Specifically, the DNA backbone channel was first extracted, and intensities were normalized using the "Normalize Local Contrast" function of ImageJ (settings: block radius = 30nm, standard deviation = 90nm, center stretch enabled). Following normalization, Otsu's method for binarization [9] and "Non-local Means De-noising"[10] (settings: sigma = 90nm, smoothing factor = 1) were applied. A morphological closing operation (radius 156 nm) was then performed to refine the segmented structures. The resulting DNA masks were skeletonized, and short skeleton fragments smaller than 420nm were excluded. NET filaments were subsequently converted into ROIs and exported. Signal intensities were measured along the line intensity profiles in all channels and exported individually for each image. Parameter files are provided in the supplementary materials.

Correlation analysis

The auto- and cross-correlation analysis of line profiles was performed using the "Analyze-Profile" workflow from our custom-developed **NanoNET** toolbox (https://github.com/ngimber/NanoNET), running on Python 3.9.13. The **NET-Analysis** workflow of NanoNET features a graphical user interface (Figure 1) for adjusting key parameters, including pixel size, filament fragment size, number of lags, minimal filament length, and peak prominence threshold, along with options for graphical output and debugging.

Specifically, line intensity profiles obtained through the NET-Detection workflow were divided into equally sized fragments (SIM and STED: 1.5 µm, dSTORM 1.0 µm). Auto- and cross-correlation was then computed for each fragment. Measurements were repeated with multiple shifts (lags) introduced for analysis. Correlation values range from -1 (indicating perfect anti-correlation) to 1 (indicating perfect correlation). Shifts corresponding to the predominant periodicity of the sample enhance the correlation, compared to arbitrary shifts. As a result, the predominant periodicities can be identified by locating the first peak in the correlation plot (correlation versus lag; Figure 1, S2). The first peak represents the predominant periodicity of the sample, and following peaks represent integer multiples of the primary periodicity, providing insights into higher-order periodic patterns within the sample. Correlation profiles were smoothed using Gaussian kernel, with a window size corresponding to the microscope resolution. Peaks were then detected using SciPy find peaks with a minimum prominence threshold of 0.4. For each protein target, we analyzed the correlation plot of 40,000–100,000 line profile fragments, calculated the first peak, and plotted those in a correlation histogram (bin size corresponds to image pixel size), enabling statistical comparisons between experimental groups. Data outputs of NanoNET included comprehensive correlation result tables, averaged correlation profiles, and lists of extracted periodicities. Parameter files are provided in the supplementary materials. For colocalization analysis, the correlation value at lag = 0 was used and plotted as violin plots (Supporting Figure S4 and S11). Analyses included data from over four biological replicates, encompassing per protein target. Gaussian peak fitting was performed in Python. Model selection between single- and two-component Gaussian fits was based on the Akaike information criterion (AIC). AIC selected single-Gaussian fits for the SIM and STED datasets, whereas only the STORM datasets were better described by bi-Gaussian fits. All graphical representations were generated using GraphPad PRISM 5. The complete analysis workflows are available as part of the NanoNET toolbox (https://github.com/ngimber/NanoNET).

Quantification of sample flatness

We used maximum intensity projections of 3D SIM/STED stacks to quantify periodicities in 2D. To test whether sample flatness could have an impact on the measured cluster distances (periodicity) after projection, we quantified the local axial tilt between neighboring clusters from 3D SIM stacks (Figure S1). Nucleosome clusters were segmented in 3D using the Blob Finder in Arivis Vision4D, and intensity-weighted centers of mass were extracted for each cluster. Nearest-neighbor pairs were identified from these coordinates, and the local axial tilt angles between neighboring clusters were computed in Python.

Auto-correlation analysis of neuronal actin

Line profile extraction and correlation analysis were performed on dSTORM images of neuronal actin using the same workflow applied to NET filaments, employing the "NET-Detection" and "NET-Analysis" functions of NanoNET. Prior to analysis, a blurred duplicate of the dSTORM reconstruction (σ = 200 nm) was added to the image stack to compensate for the lack of a continuous backbone signal and to enable robust axon detection.

SUPPLEMENTARY TABLES

Supplementary Table 1. Primary antibodies

Name	Antibody	Host	Clonality	Supplier	Catalog #	Conc. / dil. SIM & STED	Conc. / dil. STROM
**3D9**	anti-cleaved Histone 3 (3D9)	M	mAb	Brinkmann, Tilley et. al [11]	n.a.	10µg/ml	
**HNP-1**	anti-alpha Defensin 1	R	pAb	abcam	ab134706	1:50	
**CAT (D4P7B)**	Anti-catalase (D4P7B) XP®	R	mAb	Cell Signaling Technology	#12980	1:150	
**CATG (EPC)**	Anti-human Cathepsin G	R	pAb	EPC	CA617	1:250	
**NE (pAb)**	Anti-Neutrophil Elastase	R	pAb	EMD Millipore	481001	1:100	1:100
**NE (mAb)**	Anti-Neutrophil Elastase [NP57]	M	mAb	abcam	ab254178	1:50	
**Nuc (PL2.3)**	anti H2A-H2B-DNA (nucleosomes)	M	mAb	Brinkmann, Herlands et al. [12]	n.a.	2µg/ml	5µg/ml
**PR3**	Anti-Proteinase 3 (anti-serum)	R	pAb	Elastin Products	PR215	1:100	
**S100A9**	Anti-S100A9 Antibody	R	pAb	Atlas Antibodies	HPA 004193	1:20	
**TALDO1**	Anti-TALDO1	R	pAb	Atlas Antibodies	HPA 048089	1:25	

M = mouse; R = rabbit; mAb = monoclonal antibody; pAb = polyclonal antibody; conc. = concentration; dil. = dilution.

Supplementary Table 2. Secondary antibodies

Name	Fluorophore	Supplier	Catalog No	Dilution SIM	Dilution STED	Dilution STORM
anti-ms A568 F(ab')2	Alexa Fluor 568	Invitrogen	A11019	1:500	-	-
anti-rb A568	Alexa Fluor 568	Invitrogen	A-11036	1:500	-	-
anti-rb STAR ORANGE	STAR ORANGE	abberior	STORANGE	-	1:500	-
anti-ms STAR635P	STAR 635P	abberior	ST635P-1001	1:500	1:500	-
anti-ms A647 F(ab)	Alexa Fluor 647	Invitrogen	A21237	1:500	-	-
anti-rb CF680 F(ab)	CF680	Sigma	SAB4600362	-	-	1:500

Supplementary Table 3. Dyes & kits

Name	Type	Catalog #	Supplier
YOYO-1	Nucleic Acid Dye	ab275546	abcam
Phalloidin Labeling Probe Atto 488	F-actin staining	49409	Merck
Phalloidin Labeling Probe Alexa 647	F-actin staining	A22287	Thermo
CF680 Protein labelling kit	Succinimidyl ester labelling kit	92220	Biotium
Alexa647 Antibody labelling kit	Succinimidyl ester labelling kit	A88068	Thermo

REFERENCES

(1) Burn, G. L.; Raisch, T.; Tacke, S.; Winkler, M.; Prumbaum, D.; Thee, S.; Gimber, N.; Raunser, S.; Zychlinsky, A. Myeloperoxidase Transforms Chromatin into Neutrophil Extracellular Traps. *Nature* **2025**, 1–10. https://doi.org/10.1038/s41586-025-09523-9.

(2) Jhelum, H.; Čerina, D.; Harbort, C. J.; Lindner, A.; Hanitsch, L. G.; Leistner, R.; Schröder, J.-T.; von Bernuth, H.; Stegemann, M. S.; Schürmann, M.; Zychlinsky, A.; Krüger, R.; Marsman, G. Panton-Valentine Leukocidin–Induced Neutrophil Extracellular Traps Lack Antimicrobial Activity and Are Readily Induced in Patients with Recurrent PVL + - Staphylococcus Aureus Infections. *J. Leukoc. Biol.* **2024**, *115* (2), 222–234. https://doi.org/10.1093/jleuko/qiad137.

(3) Xu, K.; Zhong, G.; Zhuang, X. Actin, Spectrin, and Associated Proteins Form a Periodic Cytoskeletal Structure in Axons. *Science (1979).* **2013**, *339* (6118), 452–456. https://doi.org/10.1126/science.1232251.

(4) Kreis, P.; Gallrein, C.; Rojas-Puente, E.; Mack, T. G. A.; Kroon, C.; Dinkel, V.; Willmes, C.; Murk, K.; tom-Dieck, S.; Schuman, E. M.; Kirstein, J.; Eickholt, B. J. ATM Phosphorylation of the Actin-Binding Protein Drebrin Controls Oxidation Stress-Resistance in Mammalian Neurons and C. Elegans. *Nat. Commun.* **2019**, *10* (1), 486. https://doi.org/10.1038/s41467-019-08420-w.

(5) Ball, G.; Demmerle, J.; Kaufmann, R.; Davis, I.; Dobbie, I. M.; Schermelleh, L. SIMcheck: A Toolbox for Successful Super-Resolution Structured Illumination Microscopy. *Sci. Rep.* **2015**, *5* (1), 15915. https://doi.org/10.1038/srep15915.

(6) Gimber, N.; Strauss, S.; Jungmann, R.; Schmoranzer, J. Simultaneous Multicolor DNA-PAINT without Sequential Fluid Exchange Using Spectral Demixing. *Nano Lett.* **2022**, *22* (7), 2682–2690. https://doi.org/10.1021/acs.nanolett.1c04520.

(7) Ovesný, M.; Křížek, P.; Borkovec, J.; Švindrych, Z.; Hagen, G. M. ThunderSTORM: A Comprehensive ImageJ Plug-in for PALM and STORM Data Analysis and Super-Resolution Imaging. *Bioinformatics* **2014**, *30* (16), 2389–2390. https://doi.org/10.1093/bioinformatics/btu202.

(8) Früh, S. M.; Schoen, I.; Ries, J.; Vogel, V. Molecular Architecture of Native Fibronectin Fibrils. *Nat. Commun.* **2015**. https://doi.org/10.1038/ncomms8275.

(9) Otsu, N. A Threshold Selection Method from Gray-Level Histograms. *IEEE Trans. Syst. Man Cybern.* **1979**, *9* (1), 62–66. https://doi.org/10.1109/TSMC.1979.4310076.

(10) Buades, A.; Coll, B.; Morel, J.-M. Non-Local Means Denoising. *Image Processing On Line* **2011**, *1*, 208–212. https://doi.org/10.5201/ipol.2011.bcm_nlm.

(11) Tilley, D. O.; Abuabed, U.; Arndt, U. Z.; Schmid, M.; Florian, S.; Jungblut, P. R.; Brinkmann, V.; Herzig, A.; Zychlinsky, A. Histone H3 Clipping Is a Novel Signature of Human Neutrophil Extracellular Traps. *Elife* **2022**, *11*, 1–61. https://doi.org/10.7554/ELIFE.68283.

(12) Herlands, R. A.; William, J.; Hershberg, U.; Shlomchik, M. J. Anti-Chromatin Antibodies Drive in Vivo Antigen-Specific Activation and Somatic Hypermutation of Rheumatoid Factor B Cells at Extrafollicular Sites. *Eur. J. Immunol.* **2007**, *37* (12), 3339–3351. https://doi.org/10.1002/EJI.200737752.
